# Supplementary material for: WGS of Commensal Neisseria Reveals Acquisition of a New Ribosomal Protection Protein (MsrD) as a Possible Explanation for High Level Azithromycin Resistance in Belgium
Source: Pathogens. 2021 Mar 23;10(3):384. doi: 10.3390/pathogens10030384 (PMC8005064; doi:10.3390/pathogens10030384)

Supplemental figure 1a. Boxplot of azithromycin MICs from commensal and pathogenic isolates in the panel. Red dot indicate the median, black dots indicates all separate isolates.

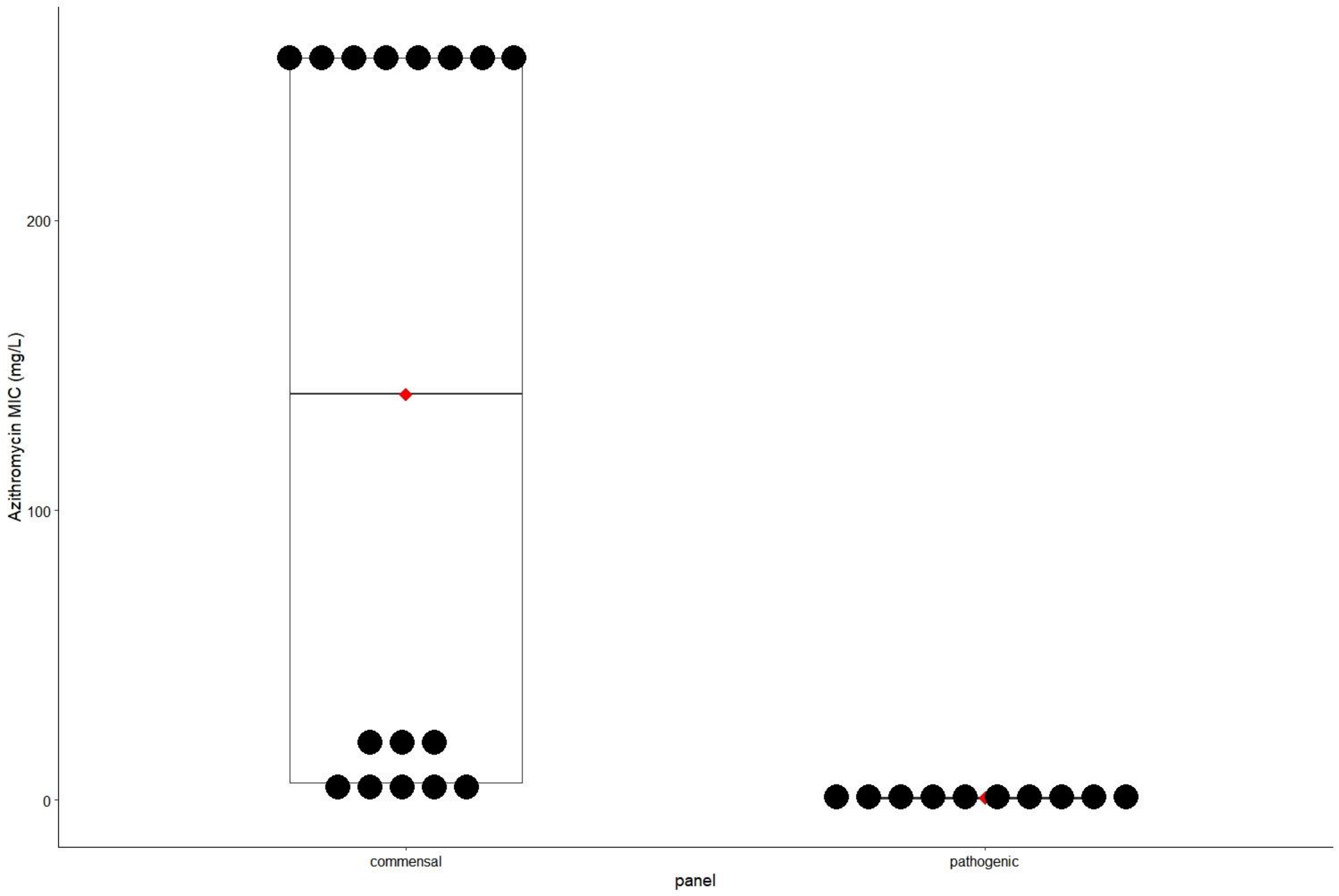

Supplement: Supplementary file 1 [file pathogens-10-00384-s001.zip › Supplemental_figure_1a_II.pdf]
