# Supplementary material for: WGS of Commensal Neisseria Reveals Acquisition of a New Ribosomal Protection Protein (MsrD) as a Possible Explanation for High Level Azithromycin Resistance in Belgium
Source: Pathogens. 2021 Mar 23;10(3):384. doi: 10.3390/pathogens10030384 (PMC8005064; doi:10.3390/pathogens10030384)
Supplement: Supplementary file 1 [file pathogens-10-00384-s001.zip › Supplemental_Figure_2.pdf]

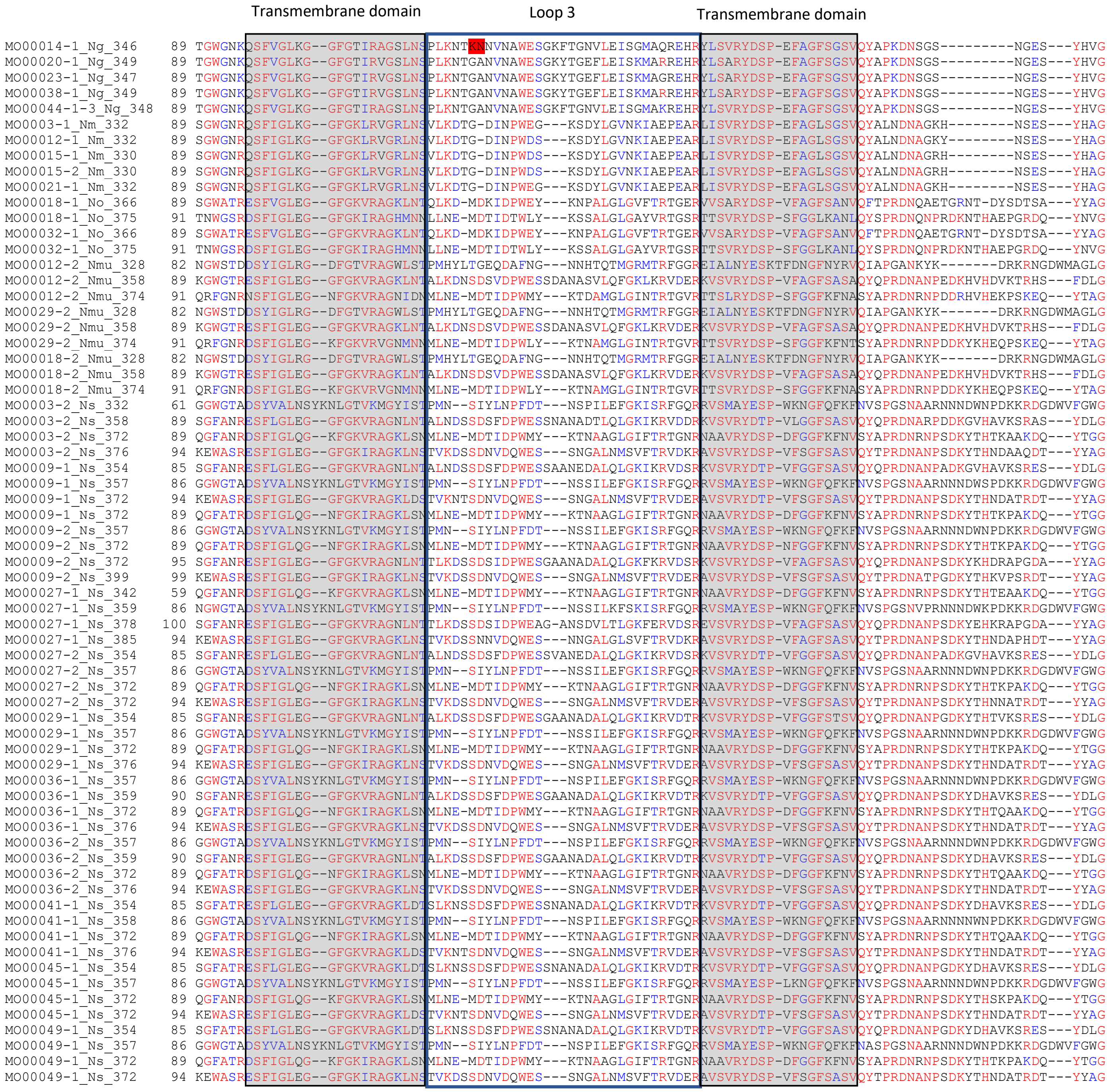

Supplemental Figure 2. Alignment of loop 3 flanking regions of *porB* paralogs found in panel. Loop 3 is boxed (blue) with resistance associated *penB* mutants in *N. gonorrhoeae* strain MO00014-1 colored in red. Transmembrane domains are shaded in grey. Strain number with species; *N. gonorrhoeae* (Ng), *N. meningitidis* (Nm), *N. oralis* (No), *N. mucosa* (Nmu) and *N. subflava* (Ns) and number of amino acids of porB.
