# Supplementary material for: WGS of Commensal Neisseria Reveals Acquisition of a New Ribosomal Protection Protein (MsrD) as a Possible Explanation for High Level Azithromycin Resistance in Belgium
Source: Pathogens. 2021 Mar 23;10(3):384. doi: 10.3390/pathogens10030384 (PMC8005064; doi:10.3390/pathogens10030384)
Supplement: Supplementary file 1 [file pathogens-10-00384-s001.zip › Supplemental_Figure_3.pdf]

```
MO0003-2           1 GTAATAATTGCACCAGTTTGAATGAAGGTAAACACATGACACTAACTAAAGCAGAATTGGCTGATATTTTGGTCGACAAA
MO0027-1           1 GTAATAATTGCACCAGTTTGAATGAAGGTAAACACATGACACTAACTAAAGCAGAATTGGCTGATATTTTGGTCGACAAA
MO00027-2          1 GTAATAATTGCACCAGTTTGAATGAAGGTAAACACATGACACTAACTAAAGCAGAATTGGCTGATATTTTGGTCGACAAA
MO0009-2           1 GTAATAATTGCACCAGTTTGAATGAAGGTAAACACATGACACTAACTAAAGCAGAATTGGCTGATATTTTGGTCGACAAA
MO00029-1          1 GTAATAATTGCACCAGTTTGAATGAAGGTAAACACATGACACTAACTAAAGCAGAATTGGCTGATATTTTGGTCGACAAA
MO00036-1          1 GTAATAATTGCACCAGTTTGAATGAAGGTAAACACATGACACTAACTAAAGCAGAATTGGCTGATATTTTGGTCGACAAA
MO00036-2          1 GTAATAATTGCACCAGTTTGAATGAAGGTAAACACATGACACTAACTAAAGCAGAATTGGCTGATATTTTGGTCGACAAA
MO00041-1          1 GTAATAATTGCACCAGTTTGAATGAAGGTAAACACATGACACTAACTAAAGCAGAATTGGCTGATATTTTGGTCGACAAA
MO00049-1          1 GTAATAATTGCACCAGTTTGAATGAAGGTAAACACATGACACTAACTAAAGCAGAATTGGCCGATATTTTGGTCGACAAA
MO0009-1           1 GTAATAATTGCACCAGTTTGAATGAAGGTAAACACATGACACTAACTAAAGCAGAATTGGCTGATATTTTGGTCGACAAA
MO00045-1          1 GTAATAATTGCACCAGTTTGAATGAAGGTAAACACATGACACTAACTAAAGCAGAATTGGCTGATATTTTGGTCGACAAA
2006-3950_Strep    1 --------------------------------------------------------------------------------
                                                               ihfA

MO0003-2          81 GTCAGCAACGTCACCAAGAATGATGCTAAAGAAATCGTCGAGCTCTTTTTTGAAGAAATCCGCAGCACTTTGGCGCGTGG
MO0027-1          81 GTCAGCAACGTCACCAAGAATGATGCTAAAGAAATCGTCGAGCTCTTTTTTGAAGAAATCCGCAGCACTTTGGCGCGTGG
MO00027-2         81 GTCAGCAACGTCACCAAGAATGATGCTAAAGAAATCGTCGAGCTCTTTTTTGAAGAAATCCGCAGCACTTTGGCGCGTGG
MO0009-2          81 GTCAGCAACGTCACCAAGAATGATGCTAAAGAAATCGTCGAGCTCTTTTTTGAAGAAATCCGCAGCACTTTGGCGCGTGG
MO00029-1         81 GTCAGCAACGTCACCAAGAATGATGCTAAAGAAATCGTCGAGCTCTTTTTTGAAGAAATCCGCAGCACTTTGGCGCGTGG
MO00036-1         81 GTCAGCAACGTCACCAAGAATGATGCTAAAGAAATCGTCGAGCTCTTTTTTGAAGAAATCCGCAGCACTTTGGCGCGTGG
MO00036-2         81 GTCAGCAACGTCACCAAGAATGATGCTAAAGAAATCGTCGAGCTCTTTTTTGAAGAAATCCGCAGCACTTTGGCGCGTGG
MO00041-1         81 GTCAGCAACGTCACCAAGAATGATGCTAAAGAAATCGTCGAGCTCTTTTTTGAAGAAATCCGCAGCACTTTGGCGCGTGG
MO00049-1         81 GTCAGCAACGTCACCAAAAATGATGCCAAAGAAATCGTCGAGCTCTTTTTTGAAGAAATCCGCAGCACTTTGGCGCGTGG
MO0009-1          81 GTCAGCAACGTCACCAAGAATGATGCCAAAGAAATCGTCGAGCTCTTTTTTGAAGAAATCCGCAGCACTTTGGCGCGTGG
MO00045-1         81 GTCAGCAACGTCACCAAGAATGATGCCAAAGAAATCGTCGAGCTCTTTTTTGAAGAAATCCGCAGCACTTTGGCGCGTGG
2006-3950_Strep    1 --------------------------------------------------------------------------------
                                                      ihfA

MO0003-2         161 TGAAGAAATTAAAATTTCCGGTTTCGGCAATTTCCAATTGCGCGACAAACCTCAACGCCCTGGCCGTAACCCTAAAACAG
MO0027-1         161 TGAAGAAATTAAAATTTCCGGTTTCGGCAATTTCCAATTGCGCGACAAACCTCAACGCCCTGGCCGTAACCCTAAAACAG
MO00027-2        161 TGAAGAAATTAAAATTTCCGGTTTCGGCAATTTCCAATTGCGCGACAAACCTCAACGCCCTGGCCGTAACCCTAAAACAG
MO0009-2         161 TGAAGAAATTAAAATTTCCGGTTTCGGCAATTTCCAATTGCGCGACAAACCTCAACGCCCTGGCCGTAACCCTAAAACAG
MO00029-1        161 TGAAGAAATTAAAATTTCCGGTTTCGGCAATTTCCAATTGCGCGACAAACCTCAACGCCCTGGCCGTAACCCTAAAACAG
MO00036-1        161 TGAAGAAATTAAAATTTCCGGTTTCGGCAATTTCCAATTGCGCGACAAACCTCAACGCCCTGGCCGTAACCCTAAAACAG
MO00036-2        161 TGAAGAAATTAAAATTTCCGGTTTCGGCAATTTCCAATTGCGCGACAAACCTCAACGCCCTGGCCGTAACCCTAAAACAG
MO00041-1        161 TGAAGAAATTAAAATTTCCGGTTTCGGCAATTTCCAATTGCGCGACAAACCTCAACGCCCTGGCCGTAACCCTAAAACAG
MO00049-1        161 TGAAGAAATTAAAATTTCCGGTTTCGGCAATTTCCAATTGCGCGACAAACCTCAACGCCCTGGCCGTAACCCTAAAACAG
MO0009-1         161 TGAAGAAATTAAAATCTCCGGTTTCGGCAATTTCCAATTGCGCGACAAACCTCAACGCCCTGGCCGTAACCCTAAAACAG
MO00045-1        161 TGAAGAAATTAAAATTTCCGGTTTCGGTAATTTCCAATTGCGCGACAAACCTCAACGCCCTGGCCGTAACCCTAAAACAG
2006-3950_Strep    1 --------------------------------------------------------------------------------
                                                      ihfA

MO0003-2         241 GTGAAGAAGTGCCGATTACCGCACGCCGTGTGGTAACTTTCCATGCCAGCCAAAAACTCAAAGGTATGGTGGAGCATTAC
MO0027-1         241 GTGAAGAAGTGCCGATTACCGCACGCCGTGTGGTAACTTTCCATGCCAGCCAAAAACTCAAAGGCATGGTGGAGCATTAC
MO00027-2        241 GTGAAGAAGTGCCGATTACCGCACGCCGTGTGGTAACTTTCCATGCCAGCCAAAAACTCAAAGGCATGGTGGAGCATTAC
MO0009-2         241 GTGAAGAAGTGCCGATTACCGCACGCCGTGTGGTAACTTTCCATGCCAGCCAAAAACTCAAAGGCATGGTGGAGCATTAC
MO00029-1        241 GTGAAGAAGTGCCGATTACCGCACGCCGTGTGGTAACTTTCCATGCCAGCCAAAAACTCAAAGGCATGGTGGAGCATTAC
MO00036-1        241 GTGAAGAAGTGCCGATTACCGCACGCCGTGTGGTAACTTTCCATGCCAGCCAAAAACTCAAAGGCATGGTGGAGCATTAC
MO00036-2        241 GTGAAGAAGTGCCGATTACCGCACGCCGTGTGGTAACTTTCCATGCCAGCCAAAAACTCAAAGGCATGGTGGAGCATTAC
MO00041-1        241 GTGAAGAAGTGCCGATTACCGCACGCCGTGTGGTAACTTTCCATGCCAGCCAAAAACTCAAAGGCATGGTGGAGCATTAC
MO00049-1        241 GTGAAGAAGTGCCGATTACCGCACGCCGTGTGGTAACTTTCCATGCCAGCCAAAAACTCAAAGGCATGGTGGAGCATTAC
MO0009-1         241 GCGAAGAAGTGCCGATTACCGCACGCCGTGTGGTAACTTTCCATGCCAGCCAAAAACTCAAAGGCATGGTGGAGCATTAC
MO00045-1        241 GCGAAGAAGTGCCGATTACCGCACGCCGTGTGGTAACTTTCCATGCCAGCCAAAAACTCAAAGGCATGGTGGAGCATTAC
2006-3950_Strep    1 --------------------------------------------------------------------------------
                                                        ihfA

MO0003-2         321 TATGACAAACAACAATAATCCGGTTATTCCTGCCAAGCGTTATTTTACGTTGGATGAGATGTGCCAATTGGTGCAAATCA
MO0027-1         321 TATGACAAACAACAATAATCCGGTTATTCCTGCCAAGCGTTATTTTACGTTGGATGAGATGTGCCAATTGGTGCAAATCA
MO00027-2        321 TATGACAAACAACAATAATCCGGTTATTCCTGCCAAGCGTTATTTTACGTTGGATGAGATGTGCCAATTGGTGCAAATCA
MO0009-2         321 TATGACAAACAACAATAATCCGGTTATTCCTGCCAAGCGTTATTTTACGTTGGATGAGATGTGCCAATTGGTGCAAATCA
MO00029-1        321 TATGACAAACAACAATAATCCGGTTATTCCTGCCAAGCGTTATTTTACGTTGGATGAGATGTGCCAATTGGTGCAAATCA
MO00036-1        321 TATGACAAACAACAATAATCCGGTTATTCCTGCCAAGCGTTATTTTACGTTGGATGAGATGTGCCAATTGGTGCAAATCA
MO00036-2        321 TATGACAAACAACAATAATCCGGTTATTCCTGCCAAGCGTTATTTTACGTTGGATGAGATGTGCCAATTGGTGCAAATCA
MO00041-1        321 TATGACAAACAACAATAATCCGGTTATTCCTGCCAAGCGTTATTTTACGTTGGATGAGATGTGCCAATTGGTGCAAATCA
MO00049-1        321 TATGACAAACAACAATAATCCGGTTATTCCTGCCAAGCGTTATTTTACGTTGGATGAGATGTGCCAATTGGTGCAAATCA
MO0009-1         321 TATGACAAACAACAATAATCCGGTTATTCCTGCCAAGCGTTATTTTACGTTGGATGAGATGTGCCAATTGGTGCAAATCA
MO00045-1        321 TATGACAAGCAACAATAATCCGGTTATTCCTGCCAAGCGTTATTTTACGTTGGATGAGATGTGCCAATTGGTGCAAATCA
2006-3950_Strep    1 --------------------------------------------------------------------------------
                          ihfA

MO0003-2         401 GCCCGGCTCAGTTTGCCCAATGGCAACATGAAAATAATGTTGTGATTGGATACGGTGGCGATCGATATACGCGCTCAGAT
MO0027-1         401 GCCCGGCTCAGTTTGCCCAATGGCAACATGAAAATAATGTTGTGATTGGATACGGTGGCGATCGATATACGCGCTCAGAT
MO00027-2        401 GCCCGGCTCAGTTTGCCCAATGGCAACATGAAAATAATGTTGTGATTGGATACGGTGGCGATCGATATACGCGCTCAGAT
MO0009-2         401 GCCCGGCTCAGTTTGCCCAATGGCAACATGAAAATAATGTTGTGATTGGATACGGTGGCGATCGATATACGCGCTCAGAT
MO00029-1        401 GCCCGGCTCAGTTTGCCCAATGGCAACATGAAAATAATGTTGTGATTGGATACGGTGGCGATCGATATACGCGCTCAGAT
MO00036-1        401 GCCCGGCTCAGTTTGCCCAATGGCAACATGAAAATAATGTTGTGATTGGATACGGTGGCGATCGATATACGCGCTCAGAT
MO00036-2        401 GCCCGGCTCAGTTTGCCCAATGGCAACATGAAAATAATGTTGTGATTGGATACGGTGGCGATCGATATACGCGCTCAGAT
MO00041-1        401 GCCCGGCTCAGTTTGCCCAATGGCAACATGAAAATAATGTTGTGATTGGATACGGTGGCGATCGATATACGCGCTCAGAT
MO00049-1        401 GCCCGGCTCAGTTTGCCCAATGGCAACATGAAAATAATGTTGTGATTGGATACGGTGGCGATCGATATACGCGCTCAGAT
MO0009-1         401 GTCCGGCTCAGTTTGCCCAATGGCAACATGAAAATAATGTTGTGATTGGATACGGTGGCGATAGATATACACGCTCAGAT
MO00045-1        401 GCCCGGCTCATTTTGCCCAATGGCAACATGAAAATAATGTTGTGATTGGATACGGTGGCGATCGATATACGCGCTCAGAT
2006-3950_Strep    1 --------------------------------------------------------------------------------
```

```
MO0003-2         481 GTAGTTAAGTTGTTGAAACTGAAAGATACGTTTGAACCGTATATCGATACGTTCAGCCGTGGCGCTTTAGATGCAAACGG
MO0027-1         481 GTAGTTAAGTTGTTGAAACTGAAAGATACGTTTGAACCGTATATCGATACGTTCAGCCGTGGCGCTTTAGATGCAAACGG
MO00027-2        481 GTAGTTAAGTTGTTGAAACTGAAAGATACGTTTGAACCGTATATCGATACGTTCAGCCGTGGCGCTTTAGATGCAAACGG
MO0009-2         481 GTAGTTAAGTTGTTGAAACTGAAAGATACGTTTGAACCGTATATCGATACGTTCAGCCGTGGCGCTTTAGATGCAAACGG
MO00029-1        481 GTAGTTAAGTTGTTGAAACTGAAAGATACGTTTGAACCGTATATCGATACGTTCAGCCGTGGCGCTTTAGATGCAAACGG
MO00036-1        481 GTAGTTAAGTTGTTGAAACTGAAAGATACGTTTGAACCGTATATCGATACGTTCAGCCGTGGCGCTTTAGATGCAAACGG
MO00036-2        481 GTAGTTAAGTTGTTGAAACTGAAAGATACGTTTGAACCGTATATCGATACGTTCAGCCGTGGCGCTTTAGATGCAAACGG
MO00041-1        481 GTAGTTAAGTTGTTGAAACTGAAAGATACGTTTGAACCGTATATCGATACGTTCAGCCGTGGCGCTTTAGATGCAAACGG
MO00049-1        481 GTAGTTAAGTTGTTGAAACTGAAAGATACGTTTGAACCGTATATCGATACGTTCAGCCGTGGCGCTTTAGATGCAAACGG
MO0009-1         481 GTAGTTAAGTTGTTGAAACTAAAAGATACGTTTGAACCGTATATCGATACGTTTAGCCGTGGTGCTTTGGATGCAAACGG
MO00045-1        481 GTAGTTAAGTTGTTGAAACTGAAAGATACGTTTGAACCATATATCGATACGTTCAGCCGTGGCGCTTTGGATGCAAACGG
2006-3950_Strep    1 --------------------------------------------------------------------------------

MO0003-2         561 CAATCCTGCCGCCAATGCAGAAGAAGTGCGTCATGGTCTGATGCAGGTTTTGTCTGATTTGGAGCGTCATCTGGGTTCTG
MO0027-1         561 CAATCCTGCCGCCAATGCAGAAGAAGTGCGTCATGGTCTGATGCAGGTTTTGTCTGATTTGGAGCGTCATCTGGGTTCTG
MO00027-2        561 CAATCCTGCCGCCAATGCAGAAGAAGTGCGTCATGGTCTGATGCAGGTTTTGTCTGATTTGGAGCGTCATCTGGGTTCTG
MO0009-2         561 CAATCCTGCCGCCAATGCAGAAGAAGTGCGTCATGGTCTGATGCAGGTTTTGTCTGATTTGGAGCGTCATCTGGGTTCTG
MO00029-1        561 CAATCCTGCCGCCAATGCAGAAGAAGTGCGTCATGGTCTGATGCAGGTTTTGTCTGATTTGGAGCGTCATCTGGGTTCTG
MO00036-1        561 CAATCCTGCCGCCAATGCAGAAGAAGTGCGTCATGGTCTGATGCAGGTTTTGTCTGATTTGGAGCGTCATCTGGGTTCTG
MO00036-2        561 CAATCCTGCCGCCAATGCAGAAGAAGTGCGTCATGGTCTGATGCAGGTTTTGTCTGATTTGGAGCGTCATCTGGGTTCTG
MO00041-1        561 CAATCCTGCCGCCAATGCAGAAGAAGTGCGTCATGGTCTGATGCAGGTTTTGTCTGATTTGGAGCGTCATCTGGGTTCTG
MO00049-1        561 CAATCCTGCCGCCAATGCAGAAGAAGTGCGTCATGGTCTGATGCAGGTTTTGTCTGATTTGGAGCGTCATCTGGGTTCTG
MO0009-1         561 CAATCCTGCCGCCAATGCAGAAGAAGTCCGTCATGGTCTGACGCAGATTTTGTCTGATTTGGAGCGTCATCTTGGTTCTG
MO00045-1        561 CAATCCTGCCGCCAATGCAGAAGAAGTCCGTCATGGTCTGATGCAGATTTTGTCTGATTTGGAGCGTCATCTTGGTTCTG
2006-3950_Strep    1 --------------------------------------------------------------------------------

MO0003-2         641 AACAACAAAATCATCATGTCGAGCAATAATCGATAAGGAAATTCTGATCAAAAGTGCGAAGGCATACAAAGATTTGCATA
MO0027-1         641 AACAACAAAATCATCATGTCGAGCAATAATCGATAAGGAAATTCTGATCAAAAGTGCGAAGGCATACAAAGATTTGCATA
MO00027-2        641 AACAACAAAATCATCATGTCGAGCAATAATCGATAAGGAAATTCTGATCAAAAGTGCGAAGGCATACAAAGATTTGCATA
MO0009-2         641 AACAACAAAATCATCATGTCGAGCAATAATCGATAAGGAAATTCTGATCAAAAGTGCGAAGGCATACAAAGATTTGCATA
MO00029-1        641 AACAACAAAATCATCATGTCGAGCAATAATCGATAAGGAAATTCTGATCAAAAGTGCGAAGGCATACAAAGATTTGCATA
MO00036-1        641 AACAACAAAATCATCATGTCGAGCAATAATCGATAAGGAAATTCTGATCAAAAGTGCGAAGGCATACAAAGATTTGCATA
MO00036-2        641 AACAACAAAATCATCATGTCGAGCAATAATCGATAAGGAAATTCTGATCAAAAGTGCGAAGGCATACAAAGATTTGCATA
MO00041-1        641 AACAACAAAATCATCATGTCGAGCAATAATCGATAAGGAAATTCTGATCAAAAGTGCGAAGGCATACAAAGATTTGCATA
MO00049-1        641 AACAACAAAATCATCATGTCGAGCAATAATCGATAAGGAAATTCTGATCAAAAGTGCGAAGGCATACAAAGATTTGCATA
MO0009-1         641 AACAACAAAACCACCATGTCGAGCTGTAATCGATAGGGAAATTTTGATCAAAAGTGCGAAGGCATATAAAGATTTGCATA
MO00045-1        641 AACAACAAAATCACCATGTCGAGCTATAATCGATAAGGAAATTCTGATCAAAAGTGCGAAGACATACAAAGATTTGCATA
2006-3950_Strep    1 --------------------------------------------------------------------------------

MO0003-2         721 AATGGGGACCAGTTTAGCCGTTTCGGGAATACTTCCTCCAAATGGATTTGTAATATTCGTAGTTTGCTGTGCAATAATGG
MO0027-1         721 AATGGGGACCAGTTTAGCCGTTTCGGGAATACTTCCTCCAAATGGATTTGTAATATTCGTAGTTTGCTGTGCAATAATGG
MO00027-2        721 AATGGGGACCAGTTTAGCCGTTTCGGGAATACTTCCTCCAAATGGATTTGTAATATTCGTAGTTTGCTGTGCAATAATGG
MO0009-2         721 AATGGGGACCAGTTTAGCCGTTTCGGGAATACTTCCTCCAAATGGATTTGTAATATTCGTAGTTTGCTGTGCAATAATGG
MO00029-1        721 AATGGGGACCAGTTTAGCCGTTTCGGGAATACTTCCTCCAAATGGATTTGTAATATTCGTAGTTTGCTGTGCAATAATGG
MO00036-1        721 AATGGGGACCAGTTTAGCCGTTTCGGGAATACTTCCTCCAAATGGATTTGTAATATTCGTAGTTTGCTGTGCAATAATGG
MO00036-2        721 AATGGGGACCAGTTTAGCCGTTTCGGGAATACTTCCTCCAAATGGATTTGTAATATTCGTAGTTTGCTGTGCAATAATGG
MO00041-1        721 AATGGGGACCAGTTTAGCCGTTTCGGGAATACTTCCTCCAAATGGATTTGTAATATTCGTAGTTTGCTGTGCAATAATGG
MO00049-1        721 AATGGGGACCAGTTTAGCCGTTTCGGGAATACTTCCTCCAAATGGATTTGTAATATTCGTAGTTTGCTGTGCAATAATGG
MO0009-1         721 --------------------------------------------------------------------------------
MO00045-1        721 --------------------------------------------------------------------------------
2006-3950_Strep    1 AATGGGGACCAGTTTAGCCGTTTCGGGAATACTTCCTCCAAATGGATTTGTAATATTCGTAGTTTGCTGTGCAATAATGG
                                                   mefA 880-1218

MO0003-2         801 GGCTTTCGGTGCCATTTTATAGCGGTGTGCAAACAGCTCTTTTTCAGGAGAAAATTAAGCCTGAATATTTAGGACGTGTA
MO0027-1         801 GGCTTTCGGTGCCATTTTATAGCGGTGTGCAAACAGCTCTTTTTCAGGAGAAAATTAAGCCTGAATATTTAGGACGTGTA
MO00027-2        801 GGCTTTCGGTGCCATTTTATAGCGGTGTGCAAACAGCTCTTTTTCAGGAGAAAATTAAGCCTGAATATTTAGGACGTGTA
MO0009-2         801 GGCTTTCGGTGCCATTTTATAGCGGTGTGCAAACAGCTCTTTTTCAGGAGAAAATTAAGCCTGAATATTTAGGACGTGTA
MO00029-1        801 GGCTTTCGGTGCCATTTTATAGCGGTGTGCAAACAGCTCTTTTTCAGGAGAAAATTAAGCCTGAATATTTAGGACGTGTA
MO00036-1        801 GGCTTTCGGTGCCATTTTATAGCGGTGTGCAAACAGCTCTTTTTCAGGAGAAAATTAAGCCTGAATATTTAGGACGTGTA
MO00036-2        801 GGCTTTCGGTGCCATTTTATAGCGGTGTGCAAACAGCTCTTTTTCAGGAGAAAATTAAGCCTGAATATTTAGGACGTGTA
MO00041-1        801 GGCTTTCGGTGCCATTTTATAGCGGTGTGCAAACAGCTCTTTTTCAGGAGAAAATTAAGCCTGAATATTTAGGACGTGTA
MO00049-1        801 GGCTTTCGGTGCCATTTTATAGCGGTGTGCAAACAGCTCTTTTTCAGGAGAAAATTAAGCCTGAATATTTAGGACGTGTA
MO0009-1         721 --------------------------------------------------------------------------------
MO00045-1        721 --------------------------------------------------------------------------------
2006-3950_Strep   81 GGCTTTCGGTGCCATTTTATAGCGGTGTGCAAACAGCTCTTTTTCAGGAGAAAATTAAGCCTGAATATTTAGGACGTGTA
                                                   mefA 880-1218

MO0003-2         881 TTTTCTTTGATCGGAAGTATCATGTCACTTGCTATGCCAATTGGGTTAATTCTTTCTGGATTCTTTGCTGATAAAATCGG
MO0027-1         881 TTTTCTTTGATCGGAAGTATCATGTCACTTGCTATGCCAATTGGGTTAATTCTTTCTGGATTCTTTGCTGATAAAATCGG
MO00027-2        881 TTTTCTTTGATCGGAAGTATCATGTCACTTGCTATGCCAATTGGGTTAATTCTTTCTGGATTCTTTGCTGATAAAATCGG
MO0009-2         881 TTTTCTTTGATCGGAAGTATCATGTCACTTGCTATGCCAATTGGGTTAATTCTTTCTGGATTCTTTGCTGATAAAATCGG
MO00029-1        881 TTTTCTTTGATCGGAAGTATCATGTCACTTGCTATGCCAATTGGGTTAATTCTTTCTGGATTCTTTGCTGATAAAATCGG
MO00036-1        881 TTTTCTTTGATCGGAAGTATCATGTCACTTGCTATGCCAATTGGGTTAATTCTTTCTGGATTCTTTGCTGATAAAATCGG
MO00036-2        881 TTTTCTTTGATCGGAAGTATCATGTCACTTGCTATGCCAATTGGGTTAATTCTTTCTGGATTCTTTGCTGATAAAATCGG
MO00041-1        881 TTTTCTTTGATCGGAAGTATCATGTCACTTGCTATGCCAATTGGGTTAATTCTTTCTGGATTCTTTGCTGATAAAATCGG
MO00049-1        881 TTTTCTTTGATCGGAAGTATCATGTCACTTGCTATGCCAATTGGGTTAATTCTTTCTGGATTCTTTGCTGATAAAATCGG
MO0009-1         721 --------------------------------------------------------------------------------
MO00045-1        721 --------------------------------------------------------------------------------
2006-3950_Strep  161 TTTTCTTTGATCGGAAGTATCATGTCACTTGCTATGCCAATTGGGTTAATTCTTTCTGGATTCTTTGCTGATAAAATCGG
                                                   mefA 880-1218
```

```
MO0003-2          961 TGTAAATCATTGGTTTTTACTATCAGGTATTTTAATTATTGGCATTGCTATAGTTTGCCAAATGATAACTGAGGTTAGAA
MO0027-1          961 TGTAAATCATTGGTTTTTACTATCAGGTATTTTAATTATTGGCATTGCTATAGTTTGCCAAATGATAACTGAGGTTAGAA
MO00027-2         961 TGTAAATCATTGGTTTTTACTATCAGGTATTTTAATTATTGGCATTGCTATAGTTTGCCAAATGATAACTGAGGTTAGAA
MO0009-2          961 TGTAAATCATTGGTTTTTACTATCAGGTATTTTAATTATTGGCATTGCTATAGTTTGCCAAATGATAACTGAGGTTAGAA
MO00029-1         961 TGTAAATCATTGGTTTTTACTATCAGGTATTTTAATTATTGGCATTGCTATAGTTTGCCAAATGATAACTGAGGTTAGAA
MO00036-1         961 TGTAAATCATTGGTTTTTACTATCAGGTATTTTAATTATTGGCATTGCTATAGTTTGCCAAATGATAACTGAGGTTAGAA
MO00036-2         961 TGTAAATCATTGGTTTTTACTATCAGGTATTTTAATTATTGGCATTGCTATAGTTTGCCAAATGATAACTGAGGTTAGAA
MO00041-1         961 TGTAAATCATTGGTTTTTACTATCAGGTATTTTAATTATTGGCATTGCTATAGTTTGCCAAATGATAACTGAGGTTAGAA
MO00049-1         961 TGTAAATCATTGGTTTTTACTATCAGGTATTTTAATTATTGGCATTGCTATAGTTTGCCAAATGATAACTGAGGTTAGAA
MO0009-1          721 --------------------------------------------------------------------------------
MO00045-1         721 --------------------------------------------------------------------------------
2006-3950_Strep   241 TGTAAATCATTGGTTTTTACTATCAGGTATTTTAATTATTGGCATTGCTATAGTTTGCCAAATGATAACTGAGGTTAGAA
                                                         mefA 880-1218

MO0003-2         1041 AATTAGATTTAAAATAAACAATATTGGAGGAATATTTATGTATCTTATTTTCATGTAACTCTTCCTGCTAAAATCGCAGG
MO0027-1         1041 AATTAGATTTAAAATAAACAATATTGGAGGAATATTTATGTATCTTATTTTCATGTAACTCTTCCTGCTAAAATCGCAGG
MO00027-2        1041 AATTAGATTTAAAATAAACAATATTGGAGGAATATTTATGTATCTTATTTTCATGTAACTCTTCCTGCTAAAATCGCAGG
MO0009-2         1041 AATTAGATTTAAAATAAACAATATTGGAGGAATATTTATGTATCTTATTTTCATGTAACTCTTCCTGCTAAAATCGCAGG
MO00029-1        1041 AATTAGATTTAAAATAAACAATATTGGAGGAATATTTATGTATCTTATTTTCATGTAACTCTTCCTGCTAAAATCGCAGG
MO00036-1        1041 AATTAGATTTAAAATAAACAATATTGGAGGAATATTTATGTATCTTATTTTCATGTAACTCTTCCTGCTAAAATCGCAGG
MO00036-2        1041 AATTAGATTTAAAATAAACAATATTGGAGGAATATTTATGTATCTTATTTTCATGTAACTCTTCCTGCTAAAATCGCAGG
MO00041-1        1041 AATTAGATTTAAAATAAACAATATTGGAGGAATATTTATGTATCTTATTTTCATGTAACTCTTCCTGCTAAAATCGCAGG
MO00049-1        1041 AATTAGATTTAAAATAAACAATATTGGAGGAATATTTATGTATCTTATTTTCATGTAACTCTTCCTGCTAAAATCGCAGG
MO0009-1          721 --------------------------------------------------------------------------------
MO00045-1         721 --------------------------------------------------------------------------------
2006-3950_Strep   321 AATTAGATTTAAAATAAACAATATTGGAGGAATATTTATGTATCTTATTTTCATGTAACTCTTCCTGCTAAAATCGCAGG
                        mefA 880-1218          Regulatory sequence motif MYLIMF

MO0003-2         1121 GTTTTCCCTGCATACAAGCAAATGAAAGCATGCGATTATAGACAGGAGGAAATGTTATGGAATTAATATTAAAAGCAAAA
MO0027-1         1121 GTTTTCCCTGCATACAAGCAAATGAAAGCATGCGATTATAGACAGGAGGAAATGTTATGGAATTAATATTAAAAGCAAAA
MO00027-2        1121 GTTTTCCCTGCATACAAGCAAATGAAAGCATGCGATTATAGACAGGAGGAAATGTTATGGAATTAATATTAAAAGCAAAA
MO0009-2         1121 GTTTTCCCTGCATACAAGCAAATGAAAGCATGCGATTATAGACAGGAGGAAATGTTATGGAATTAATATTAAAAGCAAAA
MO00029-1        1121 GTTTTCCCTGCATACAAGCAAATGAAAGCATGCGATTATAGACAGGAGGAAATGTTATGGAATTAATATTAAAAGCAAAA
MO00036-1        1121 GTTTTCCCTGCATACAAGCAAATGAAAGCATGCGATTATAGACAGGAGGAAATGTTATGGAATTAATATTAAAAGCAAAA
MO00036-2        1121 GTTTTCCCTGCATACAAGCAAATGAAAGCATGCGATTATAGACAGGAGGAAATGTTATGGAATTAATATTAAAAGCAAAA
MO00041-1        1121 GTTTTCCCTGCATACAAGCAAATGAAAGCATGCGATTATAGACAGGAGGAAATGTTATGGAATTAATATTAAAAGCAAAA
MO00049-1        1121 GTTTTCCCTGCATACAAGCAAATGAAAGCATGCGATTATAGACAGGAGGAAATGTTATGGAATTAATATTAAAAGCAAAA
MO0009-1          721 --------------------------------------------------------------------------------
MO00045-1         721 --------------------------------------------------------------------------------
2006-3950_Strep   401 GTTTTCCCTGCATACAAGCAAATGAAAGCATGCGATTATAGACAGGAGGAAATGTTATGGAATTAATATTAAAAGCAAAA
                                                                                  Msr(D)

MO0003-2         1201 GACATTCGTGTGGAATTCAAAGGACGCGATGTTTTAGATATAAATGAATTAGAAGTATATGATTATGACCGTATTGGTTT
MO0027-1         1201 GACATTCGTGTGGAATTCAAAGGACGCGATGTTTTAGATATAAATGAATTAGAAGTATATGATTATGACCGTATTGGTTT
MO00027-2        1201 GACATTCGTGTGGAATTCAAAGGACGCGATGTTTTAGATATAAATGAATTAGAAGTATATGATTATGACCGTATTGGTTT
MO0009-2         1201 GACATTCGTGTGGAATTCAAAGGACGCGATGTTTTAGATATAAATGAATTAGAAGTATATGATTATGACCGTATTGGTTT
MO00029-1        1201 GACATTCGTGTGGAATTCAAAGGACGCGATGTTTTAGATATAAATGAATTAGAAGTATATGATTATGACCGTATTGGTTT
MO00036-1        1201 GACATTCGTGTGGAATTCAAAGGACGCGATGTTTTAGATATAAATGAATTAGAAGTATATGATTATGACCGTATTGGTTT
MO00036-2        1201 GACATTCGTGTGGAATTCAAAGGACGCGATGTTTTAGATATAAATGAATTAGAAGTATATGATTATGACCGTATTGGTTT
MO00041-1        1201 GACATTCGTGTGGAATTCAAAGGACGCGATGTTTTAGATATAAATGAATTAGAAGTATATGATTATGACCGTATTGGTTT
MO00049-1        1201 GACATTCGTGTGGAATTCAAAGGACGCGATGTTTTAGATATAAATGAATTAGAAGTATATGATTATGACCGTATTGGTTT
MO0009-1          721 --------------------------------------------------------------------------------
MO00045-1         721 --------------------------------------------------------------------------------
2006-3950_Strep   481 GACATTCGTGTGGAATTCAAAGGACGCGATGTTTTAGATATAAATGAATTAGAAGTATATGATTATGACCGTATTGGTTT
                                                         Msr(D)

MO0003-2         1281 AGTAGGAGCAAATGGTGCTGGAAAAAGCACTTTACTCAGGGTACTTTTAGGAGAATTAACTCCCCCAGGATGTAAAATGA
MO0027-1         1281 AGTAGGAGCAAATGGTGCTGGAAAAAGCACTTTACTCAGGGTACTTTTAGGAGAATTAACTCCCCCAGGATGTAAAATGA
MO00027-2        1281 AGTAGGAGCAAATGGTGCTGGAAAAAGCACTTTACTCAGGGTACTTTTAGGAGAATTAACTCCCCCAGGATGTAAAATGA
MO0009-2         1281 AGTAGGAGCAAATGGTGCTGGAAAAAGCACTTTACTCAGGGTACTTTTAGGAGAATTAACTCCCCCAGGATGTAAAATGA
MO00029-1        1281 AGTAGGAGCAAATGGTGCTGGAAAAAGCACTTTACTCAGGGTACTTTTAGGAGAATTAACTCCCCCAGGATGTAAAATGA
MO00036-1        1281 AGTAGGAGCAAATGGTGCTGGAAAAAGCACTTTACTCAGGGTACTTTTAGGAGAATTAACTCCCCCAGGATGTAAAATGA
MO00036-2        1281 AGTAGGAGCAAATGGTGCTGGAAAAAGCACTTTACTCAGGGTACTTTTAGGAGAATTAACTCCCCCAGGATGTAAAATGA
MO00041-1        1281 AGTAGGAGCAAATGGTGCTGGAAAAAGCACTTTACTCAGGGTACTTTTAGGAGAATTAACTCCCCCAGGATGTAAAATGA
MO00049-1        1281 AGTAGGAGCAAATGGTGCTGGAAAAAGCACTTTACTCAGGGTACTTTTAGGAGAATTAACTCCCCCAGGATGTAAAATGA
MO0009-1          721 --------------------------------------------------------------------------------
MO00045-1         721 --------------------------------------------------------------------------------
2006-3950_Strep   561 AGTAGGAGCAAATGGTGCTGGAAAAAGCACTTTACTCAGGGTACTTTTAGGAGAATTAACTCCCCCAGGATGTAAAATGA
                                                         Msr(D)

MO0003-2         1361 ATCGTCTGGGTGAACTTGCCTATATTCCCCAGTTGGACGAAGTAACTCTGCAGGAGGAAAAAGATTTTGCACTTGTAGGC
MO0027-1         1361 ATCGTCTGGGTGAACTTGCCTATATTCCCCAGTTGGACGAAGTAACTCTGCAGGAGGAAAAAGATTTTGCACTTGTAGGC
MO00027-2        1361 ATCGTCTGGGTGAACTTGCCTATATTCCCCAGTTGGACGAAGTAACTCTGCAGGAGGAAAAAGATTTTGCACTTGTAGGC
MO0009-2         1361 ATCGTCTGGGTGAACTTGCCTATATTCCCCAGTTGGACGAAGTAACTCTGCAGGAGGAAAAAGATTTTGCACTTGTAGGC
MO00029-1        1361 ATCGTCTGGGTGAACTTGCCTATATTCCCCAGTTGGACGAAGTAACTCTGCAGGAGGAAAAAGATTTTGCACTTGTAGGC
MO00036-1        1361 ATCGTCTGGGTGAACTTGCCTATATTCCCCAGTTGGACGAAGTAACTCTGCAGGAGGAAAAAGATTTTGCACTTGTAGGC
MO00036-2        1361 ATCGTCTGGGTGAACTTGCCTATATTCCCCAGTTGGACGAAGTAACTCTGCAGGAGGAAAAAGATTTTGCACTTGTAGGC
MO00041-1        1361 ATCGTCTGGGTGAACTTGCCTATATTCCCCAGTTGGACGAAGTAACTCTGCAGGAGGAAAAAGATTTTGCACTTGTAGGC
MO00049-1        1361 ATCGTCTGGGTGAACTTGCCTATATTCCCCAGTTGGACGAAGTAACTCTGCAGGAGGAAAAAGATTTTGCACTTGTAGGC
MO0009-1          721 --------------------------------------------------------------------------------
MO00045-1         721 --------------------------------------------------------------------------------
2006-3950_Strep   641 ATCGTCTGGGTGAACTTGCCTATATTCCCCAGTTGGACGAAGTAACTCTGCAGGAGGAAAAAGATTTTGCACTTGTAGGC
                                                         Msr(D)
```

```
MO0003-2        1441 AAGCTAGGTGTTGAGCAATTAAATATACAGACTATGAGCGGTGGTGAAGAAACAAGGCTTAAAATAGCACAGGCCTTATC
MO0027-1        1441 AAGCTAGGTGTTGAGCAATTAAATATACAGACTATGAGCGGTGGTGAAGAAACAAGGCTTAAAATAGCACAGGCCTTATC
MO00027-2       1441 AAGCTAGGTGTTGAGCAATTAAATATACAGACTATGAGCGGTGGTGAAGAAACAAGGCTTAAAATAGCACAGGCCTTATC
MO0009-2        1441 AAGCTAGGTGTTGAGCAATTAAATATACAGACTATGAGCGGTGGTGAAGAAACAAGGCTTAAAATAGCACAGGCCTTATC
MO00029-1       1441 AAGCTAGGTGTTGAGCAATTAAATATACAGACTATGAGCGGTGGTGAAGAAACAAGGCTTAAAATAGCACAGGCCTTATC
MO00036-1       1441 AAGCTAGGTGTTGAGCAATTAAATATACAGACTATGAGCGGTGGTGAAGAAACAAGGCTTAAAATAGCACAGGCCTTATC
MO00036-2       1441 AAGCTAGGTGTTGAGCAATTAAATATACAGACTATGAGCGGTGGTGAAGAAACAAGGCTTAAAATAGCACAGGCCTTATC
MO00041-1       1441 AAGCTAGGTGTTGAGCAATTAAATATACAGACTATGAGCGGTGGTGAAGAAACAAGGCTTAAAATAGCACAGGCCTTATC
MO00049-1       1441 AAGCTAGGTGTTGAGCAATTAAATATACAGACTATGAGCGGTGGTGAAGAAACAAGGCTTAAAATAGCACAGGCCTTATC
MO0009-1         721 --------------------------------------------------------------------------------
MO00045-1        721 --------------------------------------------------------------------------------
2006-3950_Strep  721 AAGCTAGGTGTTGAGCAATTAAATATACAGACTATGAGCGGTGGTGAAGAAACAAGGCTTAAAATAGCACAGGCCTTATC
```

Msr(D)

```
MO0003-2        1521 GGCACAGGTTCATGGTATTTTAGCGGATGAACCTACGAGCCATTTAGACCGTGAAGGAATTGATTTTCTAATAGGACAGC
MO0027-1        1521 GGCACAGGTTCATGGTATTTTAGCGGATGAACCTACGAGCCATTTAGACCGTGAAGGAATTGATTTTCTAATAGGACAGC
MO00027-2       1521 GGCACAGGTTCATGGTATTTTAGCGGATGAACCTACGAGCCATTTAGACCGTGAAGGAATTGATTTTCTAATAGGACAGC
MO0009-2        1521 GGCACAGGTTCATGGTATTTTAGCGGATGAACCTACGAGCCATTTAGACCGTGAAGGAATTGATTTTCTAATAGGACAGC
MO00029-1       1521 GGCACAGGTTCATGGTATTTTAGCGGATGAACCTACGAGCCATTTAGACCGTGAAGGAATTGATTTTCTAATAGGACAGC
MO00036-1       1521 GGCACAGGTTCATGGTATTTTAGCGGATGAACCTACGAGCCATTTAGACCGTGAAGGAATTGATTTTCTAATAGGACAGC
MO00036-2       1521 GGCACAGGTTCATGGTATTTTAGCGGATGAACCTACGAGCCATTTAGACCGTGAAGGAATTGATTTTCTAATAGGACAGC
MO00041-1       1521 GGCACAGGTTCATGGTATTTTAGCGGATGAACCTACGAGCCATTTAGACCGTGAAGGAATTGATTTTCTAATAGGACAGC
MO00049-1       1521 GGCACAGGTTCATGGTATTTTAGCGGATGAACCTACGAGCCATTTAGACCGTGAAGGAATTGATTTTCTAATAGGACAGC
MO0009-1         721 --------------------------------------------------------------------------------
MO00045-1        721 --------------------------------------------------------------------------------
2006-3950_Strep  801 GGCACAGGTTCATGGTATTTTAGCGGATGAACCTACGAGCCATTTAGACCGTGAAGGAATTGATTTTCTAATAGGACAGC
```

Msr(D)

```
MO0003-2        1601 TAAAATATTTTACAGGTGCACTGTTAGTTATTAGCCATGACCGCTATTTTCTTGATGAAATAGTAGATAAAATATGGGAA
MO0027-1        1601 TAAAATATTTTACAGGTGCACTGTTAGTTATTAGCCATGACCGCTATTTTCTTGATGAAATAGTAGATAAAATATGGGAA
MO00027-2       1601 TAAAATATTTTACAGGTGCACTGTTAGTTATTAGCCATGACCGCTATTTTCTTGATGAAATAGTAGATAAAATATGGGAA
MO0009-2        1601 TAAAATATTTTACAGGTGCACTGTTAGTTATTAGCCATGACCGCTATTTTCTTGATGAAATAGTAGATAAAATATGGGAA
MO00029-1       1601 TAAAATATTTTACAGGTGCACTGTTAGTTATTAGCCATGACCGCTATTTTCTTGATGAAATAGTAGATAAAATATGGGAA
MO00036-1       1601 TAAAATATTTTACAGGTGCACTGTTAGTTATTAGCCATGACCGCTATTTTCTTGATGAAATAGTAGATAAAATATGGGAA
MO00036-2       1601 TAAAATATTTTACAGGTGCACTGTTAGTTATTAGCCATGACCGCTATTTTCTTGATGAAATAGTAGATAAAATATGGGAA
MO00041-1       1601 TAAAATATTTTACAGGTGCACTGTTAGTTATTAGCCATGACCGCTATTTTCTTGATGAAATAGTAGATAAAATATGGGAA
MO00049-1       1601 TAAAATATTTTACAGGTGCACTGTTAGTTATTAGCCATGACCGCTATTTTCTTGATGAAATAGTAGATAAAATATGGGAA
MO0009-1         721 --------------------------------------------------------------------------------
MO00045-1        721 --------------------------------------------------------------------------------
2006-3950_Strep  881 TAAAATATTTTACAGGTGCACTGTTAGTTATTAGCCATGACCGCTATTTTCTTGATGAAATAGTAGATAAAATATGGGAA
```

Msr(D)

```
MO0003-2        1681 CTGAAAGATGGCAAAATCACTGAGTATTGGGGAAACTATTCTGATTATCTTCGTCAGAAAGAGGAAGAACGTAAGAGCCA
MO0027-1        1681 CTGAAAGATGGCAAAATCACTGAGTATTGGGGAAACTATTCTGATTATCTTCGTCAGAAAGAGGAAGAACGTAAGAGCCA
MO00027-2       1681 CTGAAAGATGGCAAAATCACTGAGTATTGGGGAAACTATTCTGATTATCTTCGTCAGAAAGAGGAAGAACGTAAGAGCCA
MO0009-2        1681 CTGAAAGATGGCAAAATCACTGAGTATTGGGGAAACTATTCTGATTATCTTCGTCAGAAAGAGGAAGAACGTAAGAGCCA
MO00029-1       1681 CTGAAAGATGGCAAAATCACTGAGTATTGGGGAAACTATTCTGATTATCTTCGTCAGAAAGAGGAAGAACGTAAGAGCCA
MO00036-1       1681 CTGAAAGATGGCAAAATCACTGAGTATTGGGGAAACTATTCTGATTATCTTCGTCAGAAAGAGGAAGAACGTAAGAGCCA
MO00036-2       1681 CTGAAAGATGGCAAAATCACTGAGTATTGGGGAAACTATTCTGATTATCTTCGTCAGAAAGAGGAAGAACGTAAGAGCCA
MO00041-1       1681 CTGAAAGATGGCAAAATCACTGAGTATTGGGGAAACTATTCTGATTATCTTCGTCAGAAAGAGGAAGAACGTAAGAGCCA
MO00049-1       1681 CTGAAAGATGGCAAAATCACTGAGTATTGGGGAAACTATTCTGATTATCTTCGTCAGAAAGAGGAAGAACGTAAGAGCCA
MO0009-1         721 --------------------------------------------------------------------------------
MO00045-1        721 --------------------------------------------------------------------------------
2006-3950_Strep  961 CTGAAAGATGGCAAAATCACTGAGTATTGGGGAAACTATTCTGATTATCTTCGTCAGAAAGAGGAAGAACGTAAGAGCCA
```

Msr(D)

```
MO0003-2        1761 AGCTGCAGAATACGAACAATTTATTGCGGAACGTGCCCGATTGGAAAGGGCTGCGGAGGAAAAGCGAAAACAGGCTCGTA
MO0027-1        1761 AGCTGCAGAATACGAACAATTTATTGCGGAACGTGCCCGATTGGAAAGGGCTGCGGAGGAAAAGCGAAAACAGGCTCGTA
MO00027-2       1761 AGCTGCAGAATACGAACAATTTATTGCGGAACGTGCCCGATTGGAAAGGGCTGCGGAGGAAAAGCGAAAACAGGCTCGTA
MO0009-2        1761 AGCTGCAGAATACGAACAATTTATTGCGGAACGTGCCCGATTGGAAAGGGCTGCGGAGGAAAAGCGAAAACAGGCTCGTA
MO00029-1       1761 AGCTGCAGAATACGAACAATTTATTGCGGAACGTGCCCGATTGGAAAGGGCTGCGGAGGAAAAGCGAAAACAGGCTCGTA
MO00036-1       1761 AGCTGCAGAATACGAACAATTTATTGCGGAACGTGCCCGATTGGAAAGGGCTGCGGAGGAAAAGCGAAAACAGGCTCGTA
MO00036-2       1761 AGCTGCAGAATACGAACAATTTATTGCGGAACGTGCCCGATTGGAAAGGGCTGCGGAGGAAAAGCGAAAACAGGCTCGTA
MO00041-1       1761 AGCTGCAGAATACGAACAATTTATTGCGGAACGTGCCCGATTGGAAAGGGCTGCGGAGGAAAAGCGAAAACAGGCTCGTA
MO00049-1       1761 AGCTGCAGAATACGAACAATTTATTGCGGAACGTGCCCGATTGGAAAGGGCTGCGGAGGAAAAGCGAAAACAGGCTCGTA
MO0009-1         721 --------------------------------------------------------------------------------
MO00045-1        721 --------------------------------------------------------------------------------
2006-3950_Strep 1041 AGCTGCAGAATACGAACAATTTATTGCGGAACGTGCCCGATTGGAAAGGGCTGCGGAGGAAAAGCGAAAACAGGCTCGTA
```

Msr(D)

```
MO0003-2        1841 AAATAGAACAGAAGGCAAAAGGTTCTTCAAAGAAAAAAAGTACTGAAGACGGAGGGCGTTTAGCTCATCAAAAATCAATA
MO0027-1        1841 AAATAGAACAGAAGGCAAAAGGTTCTTCAAAGAAAAAAAGTACTGAAGACGGAGGGCGTTTAGCTCATCAAAAATCAATA
MO00027-2       1841 AAATAGAACAGAAGGCAAAAGGTTCTTCAAAGAAAAAAAGTACTGAAGACGGAGGGCGTTTAGCTCATCAAAAATCAATA
MO0009-2        1841 AAATAGAACAGAAGGCAAAAGGTTCTTCAAAGAAAAAAAGTACTGAAGACGGAGGGCGTTTAGCTCATCAAAAATCAATA
MO00029-1       1841 AAATAGAACAGAAGGCAAAAGGTTCTTCAAAGAAAAAAAGTACTGAAGACGGAGGGCGTTTAGCTCATCAAAAATCAATA
MO00036-1       1841 AAATAGAACAGAAGGCAAAAGGTTCTTCAAAGAAAAAAAGTACTGAAGACGGAGGGCGTTTAGCTCATCAAAAATCAATA
MO00036-2       1841 AAATAGAACAGAAGGCAAAAGGTTCTTCAAAGAAAAAAAGTACTGAAGACGGAGGGCGTTTAGCTCATCAAAAATCAATA
MO00041-1       1841 AAATAGAACAGAAGGCAAAAGGTTCTTCAAAGAAAAAAAGTACTGAAGACGGAGGGCGTTTAGCTCATCAAAAATCAATA
MO00049-1       1841 AAATAGAACAGAAGGCAAAAGGTTCTTCAAAGAAAAAAAGTACTGAAGACGGAGGGCGTTTAGCTCATCAAAAATCAATA
MO0009-1         721 --------------------------------------------------------------------------------
MO00045-1        721 --------------------------------------------------------------------------------
2006-3950_Strep 1121 AAATAGAACAGAAGGCAAAAGGTTCTTCAAAGAAAAAAAGTACTGAAGACGGAGGGCGTTTAGCTCATCAAAAATCAATA
```

Msr(D)

```
MO0003-2        1921 GGAAGTAAGGAAAAAAAGATGTATAATGCTGCTAAAACCCTAGAGCACAGGATTGCGGCCTTAGGAAAAGTAGAAGCTCC
MO0027-1        1921 GGAAGTAAGGAAAAAAAGATGTATAATGCTGCTAAAACCCTAGAGCACAGGATTGCGGCCTTAGGAAAAGTAGAAGCTCC
MO00027-2       1921 GGAAGTAAGGAAAAAAAGATGTATAATGCTGCTAAAACCCTAGAGCACAGGATTGCGGCCTTAGGAAAAGTAGAAGCTCC
MO0009-2        1921 GGAAGTAAGGAAAAAAAGATGTATAATGCTGCTAAAACCCTAGAGCACAGGATTGCGGCCTTAGGAAAAGTAGAAGCTCC
MO00029-1       1921 GGAAGTAAGGAAAAAAAGATGTATAATGCTGCTAAAACCCTAGAGCACAGGATTGCGGCCTTAGGAAAAGTAGAAGCTCC
MO00036-1       1921 GGAAGTAAGGAAAAAAAGATGTATAATGCTGCTAAAACCCTAGAGCACAGGATTGCGGCCTTAGGAAAAGTAGAAGCTCC
MO00036-2       1921 GGAAGTAAGGAAAAAAAGATGTATAATGCTGCTAAAACCCTAGAGCACAGGATTGCGGCCTTAGGAAAAGTAGAAGCTCC
MO00041-1       1921 GGAAGTAAGGAAAAAAAGATGTATAATGCTGCTAAAACCCTAGAGCACAGGATTGCGGCCTTAGGAAAAGTAGAAGCTCC
MO00049-1       1921 GGAAGTAAGGAAAAAAAGATGTATAATGCTGCTAAAACCCTAGAGCACAGGATTGCGGCCTTAGGAAAAGTAGAAGCTCC
MO0009-1         721 --------------------------------------------------------------------------------
MO00045-1        721 --------------------------------------------------------------------------------
2006-3950_Strep 1201 GGAAGTAAGGAAAAAAAGATGTATAATGCTGCTAAAACCCTAGAGCACAGGATTGCGGCCTTAGGAAAAGTAGAAGCTCC
                                                     Msr(D)

MO0003-2        2001 GGAAGGCATTCGCAGAATTCGTTTCAGGCAAAGTAAAGCATTGGAGCTCCATAATCCATACCCTATAGTCGGTGCAGAAA
MO0027-1        2001 GGAAGGCATTCGCAGAATTCGTTTCAGGCAAAGTAAAGCATTGGAGCTCCATAATCCATACCCTATAGTCGGTGCAGAAA
MO00027-2       2001 GGAAGGCATTCGCAGAATTCGTTTCAGGCAAAGTAAAGCATTGGAGCTCCATAATCCATACCCTATAGTCGGTGCAGAAA
MO0009-2        2001 GGAAGGCATTCGCAGAATTCGTTTCAGGCAAAGTAAAGCATTGGAGCTCCATAATCCATACCCTATAGTCGGTGCAGAAA
MO00029-1       2001 GGAAGGCATTCGCAGAATTCGTTTCAGGCAAAGTAAAGCATTGGAGCTCCATAATCCATACCCTATAGTCGGTGCAGAAA
MO00036-1       2001 GGAAGGCATTCGCAGAATTCGTTTCAGGCAAAGTAAAGCATTGGAGCTCCATAATCCATACCCTATAGTCGGTGCAGAAA
MO00036-2       2001 GGAAGGCATTCGCAGAATTCGTTTCAGGCAAAGTAAAGCATTGGAGCTCCATAATCCATACCCTATAGTCGGTGCAGAAA
MO00041-1       2001 GGAAGGCATTCGCAGAATTCGTTTCAGGCAAAGTAAAGCATTGGAGCTCCATAATCCATACCCTATAGTCGGTGCAGAAA
MO00049-1       2001 GGAAGGCATTCGCAGAATTCGTTTCAGGCAAAGTAAAGCATTGGAGCTCCATAATCCATACCCTATAGTCGGTGCAGAAA
MO0009-1         721 --------------------------------------------------------------------------------
MO00045-1        721 --------------------------------------------------------------------------------
2006-3950_Strep 1281 GGAAGGCATTCGCAGAATTCGTTTCAGGCAAAGTAAAGCATTGGAGCTCCATAATCCATACCCTATAGTCGGTGCAGAAA
                                                     Msr(D)

MO0003-2        2081 TTAATAAAGTATTTGGGGATAAGGCTCTGTTTGAAAATGCATCTTTTCAAATTCCGTTAGGAGCAAAAGTGGCGTTAACT
MO0027-1        2081 TTAATAAAGTATTTGGGGATAAGGCTCTGTTTGAAAATGCATCTTTTCAAATTCCGTTAGGAGCAAAAGTGGCGTTAACT
MO00027-2       2081 TTAATAAAGTATTTGGGGATAAGGCTCTGTTTGAAAATGCATCTTTTCAAATTCCGTTAGGAGCAAAAGTGGCGTTAACT
MO0009-2        2081 TTAATAAAGTATTTGGGGATAAGGCTCTGTTTGAAAATGCATCTTTTCAAATTCCGTTAGGAGCAAAAGTGGCGTTAACT
MO00029-1       2081 TTAATAAAGTATTTGGGGATAAGGCTCTGTTTGAAAATGCATCTTTTCAAATTCCGTTAGGAGCAAAAGTGGCGTTAACT
MO00036-1       2081 TTAATAAAGTATTTGGGGATAAGGCTCTGTTTGAAAATGCATCTTTTCAAATTCCGTTAGGAGCAAAAGTGGCGTTAACT
MO00036-2       2081 TTAATAAAGTATTTGGGGATAAGGCTCTGTTTGAAAATGCATCTTTTCAAATTCCGTTAGGAGCAAAAGTGGCGTTAACT
MO00041-1       2081 TTAATAAAGTATTTGGGGATAAGGCTCTGTTTGAAAATGCATCTTTTCAAATTCCGTTAGGAGCAAAAGTGGCGTTAACT
MO00049-1       2081 TTAATAAAGTATTTGGGGATAAGGCTCTGTTTGAAAATGCATCTTTTCAAATTCCGTTAGGAGCAAAAGTGGCGTTAACT
MO0009-1         721 --------------------------------------------------------------------------------
MO00045-1        721 --------------------------------------------------------------------------------
2006-3950_Strep 1361 TTAATAAAGTATTTGGGGATAAGGCTCTGTTTGAAAATGCATCTTTTCAAATTCCGTTAGGAGCAAAAGTGGCGTTAACT
                                                     Msr(D)

MO0003-2        2161 GGTGGTAATGGAATCGGAAAAACAACTTTAATCCAAATGATCTTAAACCATGAAGAAGGAATTTCTATTTCGCCTAAGGC
MO0027-1        2161 GGTGGTAATGGAATCGGAAAAACAACTTTAATCCAAATGATCTTAAACCATGAAGAAGGAATTTCTATTTCGCCTAAGGC
MO00027-2       2161 GGTGGTAATGGAATCGGAAAAACAACTTTAATCCAAATGATCTTAAACCATGAAGAAGGAATTTCTATTTCGCCTAAGGC
MO0009-2        2161 GGTGGTAATGGAATCGGAAAAACAACTTTAATCCAAATGATCTTAAACCATGAAGAAGGAATTTCTATTTCGCCTAAGGC
MO00029-1       2161 GGTGGTAATGGAATCGGAAAAACAACTTTAATCCAAATGATCTTAAACCATGAAGAAGGAATTTCTATTTCGCCTAAGGC
MO00036-1       2161 GGTGGTAATGGAATCGGAAAAACAACTTTAATCCAAATGATCTTAAACCATGAAGAAGGAATTTCTATTTCGCCTAAGGC
MO00036-2       2161 GGTGGTAATGGAATCGGAAAAACAACTTTAATCCAAATGATCTTAAACCATGAAGAAGGAATTTCTATTTCGCCTAAGGC
MO00041-1       2161 GGTGGTAATGGAATCGGAAAAACAACTTTAATCCAAATGATCTTAAACCATGAAGAAGGAATTTCTATTTCGCCTAAGGC
MO00049-1       2161 GGTGGTAATGGAATCGGAAAAACAACTTTAATCCAAATGATCTTAAACCATGAAGAAGGAATTTCTATTTCGCCTAAGGC
MO0009-1         721 --------------------------------------------------------------------------------
MO00045-1        721 --------------------------------------------------------------------------------
2006-3950_Strep 1441 GGTGGTAATGGAATCGGAAAAACAACTTTAATCCAAATGATCTTAAACCATGAAGAAGGAATTTCTATTTCGCCTAAGGC
                                                     Msr(D)

MO0003-2        2241 AAAAATAGGTTACTTTGCACAGAATGGTTACAAGTACAACAGTAATCAGAATGTTATGGAGTTTATGCAGAAGGATTGTG
MO0027-1        2241 AAAAATAGGTTACTTTGCACAGAATGGTTACAAGTACAACAGTAATCAGAATGTTATGGAGTTTATGCAGAAGGATTGTG
MO00027-2       2241 AAAAATAGGTTACTTTGCACAGAATGGTTACAAGTACAACAGTAATCAGAATGTTATGGAGTTTATGCAGAAGGATTGTG
MO0009-2        2241 AAAAATAGGTTACTTTGCACAGAATGGTTACAAGTACAACAGTAATCAGAATGTTATGGAGTTTATGCAGAAGGATTGTG
MO00029-1       2241 AAAAATAGGTTACTTTGCACAGAATGGTTACAAGTACAACAGTAATCAGAATGTTATGGAGTTTATGCAGAAGGATTGTG
MO00036-1       2241 AAAAATAGGTTACTTTGCACAGAATGGTTACAAGTACAACAGTAATCAGAATGTTATGGAGTTTATGCAGAAGGATTGTG
MO00036-2       2241 AAAAATAGGTTACTTTGCACAGAATGGTTACAAGTACAACAGTAATCAGAATGTTATGGAGTTTATGCAGAAGGATTGTG
MO00041-1       2241 AAAAATAGGTTACTTTGCACAGAATGGTTACAAGTACAACAGTAATCAGAATGTTATGGAGTTTATGCAGAAGGATTGTG
MO00049-1       2241 AAAAATAGGTTACTTTGCACAGAATGGTTACAAGTACAACAGTAATCAGAATGTTATGGAGTTTATGCAGAAGGATTGTG
MO0009-1         721 --------------------------------------------------------------------------------
MO00045-1        721 --------------------------------------------------------------------------------
2006-3950_Strep 1521 AAAAATAGGTTACTTTGCACAGAATGGTTACAAGTACAACAGTAATCAGAATGTTATGGAGTTTATGCAGAAGGATTGTG
                                                     Msr(D)

MO0003-2        2321 ACTACAATATATCAGAAATTCGTTCAGTGCTAGCATCTATGGGGTTCAAACAGAACGATATTGGAAAAAGTTTATCTGTT
MO0027-1        2321 ACTACAATATATCAGAAATTCGTTCAGTGCTAGCATCTATGGGGTTCAAACAGAACGATATTGGAAAAAGTTTATCTGTT
MO00027-2       2321 ACTACAATATATCAGAAATTCGTTCAGTGCTAGCATCTATGGGGTTCAAACAGAACGATATTGGAAAAAGTTTATCTGTT
MO0009-2        2321 ACTACAATATATCAGAAATTCGTTCAGTGCTAGCATCTATGGGGTTCAAACAGAACGATATTGGAAAAAGTTTATCTGTT
MO00029-1       2321 ACTACAATATATCAGAAATTCGTTCAGTGCTAGCATCTATGGGGTTCAAACAGAACGATATTGGAAAAAGTTTATCTGTT
MO00036-1       2321 ACTACAATATATCAGAAATTCGTTCAGTGCTAGCATCTATGGGGTTCAAACAGAACGATATTGGAAAAAGTTTATCTGTT
MO00036-2       2321 ACTACAATATATCAGAAATTCGTTCAGTGCTAGCATCTATGGGGTTCAAACAGAACGATATTGGAAAAAGTTTATCTGTT
MO00041-1       2321 ACTACAATATATCAGAAATTCGTTCAGTGCTAGCATCTATGGGGTTCAAACAGAACGATATTGGAAAAAGTTTATCTGTT
MO00049-1       2321 ACTACAATATATCAGAAATTCGTTCAGTGCTAGCATCTATGGGGTTCAAACAGAACGATATTGGAAAAAGTTTATCTGTT
MO0009-1         721 --------------------------------------------------------------------------------
MO00045-1        721 --------------------------------------------------------------------------------
2006-3950_Strep 1601 ACTACAATATATCAGAAATTCGTTCAGTGCTAGCATCTATGGGGTTCAAACAGAACGATATTGGAAAAAGTTTATCTGTT
                                                     Msr(D)
```

```
MO0003-2          2401 TTAAGCGGTGGAGAAATTATAAAATTGTTGCTTGCTAAAATGCTCATGGGTAGATATAACATCCTAATAATGGATGAACC
MO0027-1          2401 TTAAGCGGTGGAGAAATTATAAAATTGTTGCTTGCTAAAATGCTCATGGGTAGATATAACATCCTAATAATGGATGAACC
MO00027-2         2401 TTAAGCGGTGGAGAAATTATAAAATTGTTGCTTGCTAAAATGCTCATGGGTAGATATAACATCCTAATAATGGATGAACC
MO0009-2          2401 TTAAGCGGTGGAGAAATTATAAAATTGTTGCTTGCTAAAATGCTCATGGGTAGATATAACATCCTAATAATGGATGAACC
MO00029-1         2401 TTAAGCGGTGGAGAAATTATAAAATTGTTGCTTGCTAAAATGCTCATGGGTAGATATAACATCCTAATAATGGATGAACC
MO00036-1         2401 TTAAGCGGTGGAGAAATTATAAAATTGTTGCTTGCTAAAATGCTCATGGGTAGATATAACATCCTAATAATGGATGAACC
MO00036-2         2401 TTAAGCGGTGGAGAAATTATAAAATTGTTGCTTGCTAAAATGCTCATGGGTAGATATAACATCCTAATAATGGATGAACC
MO00041-1         2401 TTAAGCGGTGGAGAAATTATAAAATTGTTGCTTGCTAAAATGCTCATGGGTAGATATAACATCCTAATAATGGATGAACC
MO00049-1         2401 TTAAGCGGTGGAGAAATTATAAAATTGTTGCTTGCTAAAATGCTCATGGGTAGATATAACATCCTAATAATGGATGAACC
MO0009-1           721 --------------------------------------------------------------------------------
MO00045-1          721 --------------------------------------------------------------------------------
2006-3950_Strep   1681 TTAAGCGGTGGAGAAATTATAAAATTGTTGCTTGCTAAAATGCTCATGGGTAGATATAACATCCTAATAATGGATGAACC
                                                      Msr(D)

MO0003-2          2481 CAGTAACTTCCTTGACATACCAAGTTTAGAGGCTTTGGAAATACTAATGAAGGAGTACACCGGAACTATCGTGTTTATCA
MO0027-1          2481 CAGTAACTTCCTTGACATACCAAGTTTAGAGGCTTTGGAAATACTAATGAAGGAGTACACCGGAACTATCGTGTTTATCA
MO00027-2         2481 CAGTAACTTCCTTGACATACCAAGTTTAGAGGCTTTGGAAATACTAATGAAGGAGTACACCGGAACTATCGTGTTTATCA
MO0009-2          2481 CAGTAACTTCCTTGACATACCAAGTTTAGAGGCTTTGGAAATACTAATGAAGGAGTACACCGGAACTATCGTGTTTATCA
MO00029-1         2481 CAGTAACTTCCTTGACATACCAAGTTTAGAGGCTTTGGAAATACTAATGAAGGAGTACACCGGAACTATCGTGTTTATCA
MO00036-1         2481 CAGTAACTTCCTTGACATACCAAGTTTAGAGGCTTTGGAAATACTAATGAAGGAGTACACCGGAACTATCGTGTTTATCA
MO00036-2         2481 CAGTAACTTCCTTGACATACCAAGTTTAGAGGCTTTGGAAATACTAATGAAGGAGTACACCGGAACTATCGTGTTTATCA
MO00041-1         2481 CAGTAACTTCCTTGACATACCAAGTTTAGAGGCTTTGGAAATACTAATGAAGGAGTACACCGGAACTATCGTGTTTATCA
MO00049-1         2481 CAGTAACTTCCTTGACATACCAAGTTTAGAGGCTTTGGAAATACTAATGAAGGAGTACACCGGAACTATCGTGTTTATCA
MO0009-1           721 --------------------------------------------------------------------------------
MO00045-1          721 --------------------------------------------------------------------------------
2006-3950_Strep   1761 CAGTAACTTCCTTGACATACCAAGTTTAGAGGCTTTGGAAATACTAATGAAGGAGTACACCGGAACTATCGTGTTTATCA
                                                      Msr(D)

MO0003-2          2561 CCCACGATAAACGATTACTCGAAAATGTAGCAGATGTAGTTTATGAAATTAGAGATAAGAAAATAAATCTGAAACATTAA
MO0027-1          2561 CCCACGATAAACGATTACTCGAAAATGTAGCAGATGTAGTTTATGAAATTAGAGATAAGAAAATAAATCTGAAACATTAA
MO00027-2         2561 CCCACGATAAACGATTACTCGAAAATGTAGCAGATGTAGTTTATGAAATTAGAGATAAGAAAATAAATCTGAAACATTAA
MO0009-2          2561 CCCACGATAAACGATTACTCGAAAATGTAGCAGATGTAGTTTATGAAATTAGAGATAAGAAAATAAATCTGAAACATTAA
MO00029-1         2561 CCCACGATAAACGATTACTCGAAAATGTAGCAGATGTAGTTTATGAAATTAGAGATAAGAAAATAAATCTGAAACATTAA
MO00036-1         2561 CCCACGATAAACGATTACTCGAAAATGTAGCAGATGTAGTTTATGAAATTAGAGATAAGAAAATAAATCTGAAACATTAA
MO00036-2         2561 CCCACGATAAACGATTACTCGAAAATGTAGCAGATGTAGTTTATGAAATTAGAGATAAGAAAATAAATCTGAAACATTAA
MO00041-1         2561 CCCACGATAAACGATTACTCGAAAATGTAGCAGATGTAGTTTATGAAATTAGAGATAAGAAAATAAATCTGAAACATTAA
MO00049-1         2561 CCCACGATAAACGATTACTCGAAAATGTAGCAGATGTAGTTTATGAAATTAGAGATAAGAAAATAAATCTGAAACATTAA
MO0009-1           721 --------------------------------------------------------------------------------
MO00045-1          721 --------------------------------------------------------------------------------
2006-3950_Strep   1841 CCCACGATAAACGATTACTCGAAAATGTAGCAGATGTAGTTTATGAAATTAGAGATAAGAAAATAAATCTGAAACATTAA
                                                      Msr(D)

MO0003-2          2641 ATTTAAGGTAGTCGCTGGTCAGTATTGATTCTAATAAAGGCTGTTTGAATGATTTTCAGACGGCCTTTATTGTTTTATAT
MO0027-1          2641 ATTTAAGGTAGTCGCTGGTCAGTATTGATTCTAATAAAGGCTGTTTGAATGATTTTCAGACGGCCTTTATTGTTTTATAT
MO00027-2         2641 ATTTAAGGTAGTCGCTGGTCAGTATTGATTCTAATAAAGGCTGTTTGAATGATTTTCAGACGGCCTTTATTGTTTTATAT
MO0009-2          2641 ATTTAAGGTAGTCGCTGGTCAGTATTGATTCTAATAAAGGCTGTTTGAATGATTTTCAGACGGCCTTTATTGTTTTATAT
MO00029-1         2641 ATTTAAGGTAGTCGCTGGTCAGTATTGATTCTAATAAAGGCTGTTTGAATGATTTTCAGACGGCCTTTATTGTTTTATAT
MO00036-1         2641 ATTTAAGGTAGTCGCTGGTCAGTATTGATTCTAATAAAGGCTGTTTGAATGATTTTCAGACGGCCTTTATTGTTTTATAT
MO00036-2         2641 ATTTAAGGTAGTCGCTGGTCAGTATTGATTCTAATAAAGGCTGTTTGAATGATTTTCAGACGGCCTTTATTGTTTTATAT
MO00041-1         2641 ATTTAAGGTAGTCGCTGGTCAGTATTGATTCTAATAAAGGCTGTTTGAATGATTTTCAGACGGCCTTTATTGTTTTATAT
MO00049-1         2641 ATTTAAGGTAGTCGCTGGTCAGTATTGATTCTAATAAAGGCTGTTTGAATGATTTTCAGACGGCCTTTATTGTTTTATAT
MO0009-1           721 -----------------------ATTGATTCTAATAAAGGCTGTTTGAATGATTTTCAGACGGCCTTTATTGTTTTATAT
MO00045-1          721 -----------------------ATTGATTCTAATAAAGGCTGTTTGAATGATTTTCAGACGGCCTTTATTGTTTTATAT
2006-3950_Strep   1921 ATTTAAGGTAGTCGCTGGTCAGTA--------------------------------------------------------
                                                                           DUS

MO0003-2          2721 TGCTTTCAAAATATCAGAATCTTGATTGCAAATGATTCTATGAAATAGCCGTAAATGTTATACTTAGCCTCTTGTTTGCC
MO0027-1          2721 TGCTTTCAAAATATCAGAATCTTGATTGCAAATGATTCTATGAAATAGCCGTAAATGTTATACTTAGCCTCTTGTTTGCC
MO00027-2         2721 TGCTTTCAAAATATCAGAATCTTGATTGCAAATGATTCTATGAAATAGCCGTAAATGTTATACTTAGCCTCTTGTTTGCC
MO0009-2          2721 TGCTTTCAAAATATCAGAATCTTGATTGCAAATGATTCTATGAAATAGCCGTAAATGTTATACTTAGCCTCTTGTTTGCC
MO00029-1         2721 TGCTTTCAAAATATCAGAATCTTGATTGCAAATGATTCTATGAAATAGCCGTAAATGTTATACTTAGCCTCTTGTTTGCC
MO00036-1         2721 TGCTTTCAAAATATCAGAATCTTGATTGCAAATGATTCTATGAAATAGCCGTAAATGTTATACTTAGCCTCTTGTTTGCC
MO00036-2         2721 TGCTTTCAAAATATCAGAATCTTGATTGCAAATGATTCTATGAAATAGCCGTAAATGTTATACTTAGCCTCTTGTTTGCC
MO00041-1         2721 TGCTTTCAAAATATCAGAATCTTGATTGCAAATGATTCTATGAAATAGCCGTAAATGTTATACTTAGCCTCTTGTTTGCC
MO00049-1         2721 TGCTTTCAAAATATCAGAATCTTGATTGCAAATGATTCTATGAAATAGCCGTAAATGTTATACTTAGCCTCTTGTTTGCC
MO0009-1           778 TGCTTTCAAAATATCAGAATCTTGATTGCAAATGATTCTATGAAATAGCCGTAAATGTTATACTTAGCCTCTTGTTTGCC
MO00045-1          778 TGCGTTCAAAATATCAGAATCTTGATTGCAAATGATTCTATGAAATAGCCGTAAATGTTATACTTAGCCTCTTGTTTGCC
2006-3950_Strep        --------------------------------------------------------------------------------

MO0003-2          2801 GATTGGGAAGAAACAGTCATTAGATAATGCGGATAGATGCCGCCTTTTCATGGCAGACTGAGGTTTTTAAAATAAATGTA
MO0027-1          2801 GATTGGGAAGAAACAGTCATTAGATAATGCGGATAGATGCCGCCTTTTCATGGCAGACTGAGGTTTTTAAAATAAATGTA
MO00027-2         2801 GATTGGGAAGAAACAGTCATTAGATAATGCGGATAGATGCCGCCTTTTCATGGCAGACTGAGGTTTTTAAAATAAATGTA
MO0009-2          2801 GATTGGGAAGAAACAGTCATTAGATAATGCGGATAGATGCCGCCTTTTCATGGCAGACTGAGGTTTTTAAAATAAATGTA
MO00029-1         2801 GATTGGGAAGAAACAGTCATTAGATAATGCGGATAGATGCCGCCTTTTCATGGCAGACTGAGGTTTTTAAAATAAATGTA
MO00036-1         2801 GATTGGGAAGAAACAGTCATTAGATAATGCGGATAGATGCCGCCTTTTCATGGCAGACTGAGGTTTTTAAAATAAATGTA
MO00036-2         2801 GATTGGGAAGAAACAGTCATTAGATAATGCGGATAGATGCCGCCTTTTCATGGCAGACTGAGGTTTTTAAAATAAATGTA
MO00041-1         2801 GATTGGGAAGAAACAGTCATTAGATAATGCGGATAGATGCCGCCTTTTCATGGCAGACTGAGGTTTTTAAAATAAATGTA
MO00049-1         2801 GATTGGGAAGAAACAGTCATTAGATAATGCGGATGGATGCCGCCTTTTCATGGCAGACTGAGGTTTTTAAAACAAATGTA
MO0009-1           858 GATTGGGAAGAAACAGTCATTAGATAATGCGGATAAATGCCGCCTTTTCATGGCAGACTGAGGTTTTTAAAATAAATGTA
MO00045-1          858 GATTGGGAAGAAACAGTCATTGGATAATGCGGATAGATGCCGCCTTTTCATGGCAGACTGAGGTTTTTAAAACAAATGTA
2006-3950_Strep        --------------------------------------------------------------------------------
```

```
MO0003-2         2881 ACGGTAGAATAAACAGGCTGCCGGACAACATTCAAATCGATGAAATGGATAGCAGAAAATGAGCAGAATCCAACAAACTT
MO0027-1         2881 ACGGTAGAATAAACAGGCTGCCGGACAACATTCAAATCGATGAAATGGATAGCAGAAAATGAGCAGAATCCAACAAACTT
MO00027-2        2881 ACGGTAGAATAAACAGGCTGCCGGACAACATTCAAATCGATGAAATGGATAGCAGAAAATGAGCAGAATCCAACAAACTT
MO0009-2         2881 ACGGTAGAATAAACAGGCTGCCGGACAACATTCAAATCGATGAAATGGATAGCAGAAAATGAGCAGAATCCAACAAACTT
MO00029-1        2881 ACGGTAGAATAAACAGGCTGCCGGACAACATTCAAATCGATGAAATGGATAGCAGAAAATGAGCAGAATCCAACAAACTT
MO00036-1        2881 ACGGTAGAATAAACAGGCTGCCGGACAACATTCAAATCGATGAAATGGATAGCAGAAAATGAGCAGAATCCAACAAACTT
MO00036-2        2881 ACGGTAGAATAAACAGGCTGCCGGACAACATTCAAATCGATGAAATGGATAGCAGAAAATGAGCAGAATCCAACAAACTT
MO00041-1        2881 ACGGTAGAATAAACAGGCTGCCGGACAACATTCAAATCGATGAAATGGATAGCAGAAAATGAGCAGAATCCAACAAACTT
MO00049-1        2881 ACGGCAGAATAAATAGGCTGCCGGACAACATTCAAATCGATGAAATGGATAGCAGGAAATGAGCAGAATCCAACAAACTT
MO0009-1          938 ACGGCAGAATAAACAGGCTGCCGGACAACATTCAAATCGATGAAATGGATAGCAGGAAATGAGCAGAATCCAACAAACTT
MO00045-1         938 ACGGCAGCATAAACAGGCTGCCGGACAACATTCAAATCGATGAAATGGATAGCAGAAAATGAGCAGAATCCAACACACTT
2006-3950_Strep       --------------------------------------------------------------------------------
                                                                                     trpA

MO0003-2         2961 TTTCAGCGCTCAATGGCGCAAAAGCACTGATTCCTTATATTACGGTGGGTGATCCCAACCTTGAGACGACCCTGGCATTG
MO0027-1         2961 TCTCAGCGCTCAATGGCGCAAAAGCACTGATTCCTTATATTACGGTGGGTGATCCCAACCTTGAGACGACCCTGGCATTG
MO00027-2        2961 TCTCAGCGCTCAATGGCGCAAAAGCACTGATTCCTTATATTACGGTGGGTGATCCCAACCTTGAGACGACCCTGGCATTG
MO0009-2         2961 TCTCAGCGCTCAATGGCGCAAAAGCACTGATTCCTTATATTACGGTGGGTGATCCCAACCTTGAGACAACCTTGGCATTG
MO00029-1        2961 TCTCAGCGCTCAATGGCGCAAAAGCACTGATTCCTTATATTACGGTGGGTGATCCCAACCTTGAGACGACCCTGGCATTG
MO00036-1        2961 TCTCAGCGCTCAATGGCGCAAAAGCACTGATTCCTTATATTACGGTGGGTGATCCCAACCTTGAGACGACCCTGGCATTG
MO00036-2        2961 TCTCAGCGCTCAATGGCGCAAAAGCACTGATTCCTTATATTACGGTGGGTGATCCCAACCTTGAGACGACCCTGGCATTG
MO00041-1        2961 TCTCAGCGCTCAATGGCGCAAAAGCACTGATTCCTTATATTACGGTGGGTGATCCCAACCTTGAGACGACCCTGGCATTG
MO00049-1        2961 TCTCAGCGCTCAATGGCGCAAAAGCACTGATTCCTTATATTACGGTGGGCGATCCCAGCCTTGAGACAACCTTGGCATTG
MO0009-1         1018 TCTCAGCGCTCAATGGCGCAAAAGCACTGATTCCTTATATTACGGTGGGTGATCCCAGTCTTGAGACAACCTTGGCATTG
MO00045-1        1018 TCTCAGCGCTCAATGGCGCAAAAGCACTGATTCCTTATATTACGGTGGGCGATCCCAGCCTTGAGACAACCTTGGCATTG
2006-3950_Strep       --------------------------------------------------------------------------------
                                                      trpA

MO0003-2         3041 ATGCACAGCCTGGTAGCTAACGGTGCTGATATTTTGGAACTGGGTGTGCCGTTTTCTGACCCGATGGCGGATGGTCCGAC
MO0027-1         3041 ATGCACAGCCTGGTAGCTAACGGTGCTGATATTTTGGAACTGGGTGTGCCGTTTTCTGACCCGATGGCGGATGGTCCGAC
MO00027-2        3041 ATGCACAGCCTGGTAGCTAACGGTGCTGATATTTTGGAACTGGGTGTGCCGTTTTCTGACCCGATGGCGGATGGTCCGAC
MO0009-2         3041 ATGCACAGCCTGGTAGCTAACGGTGCTGATATTTTGGAACTGGGTGTGCCGTTTTCTGACCCGATGGCGGATGGTCCGAC
MO00029-1        3041 ATGCACAGCCTGGTAGCTAACGGTGCTGATATTTTGGAACTGGGTGTGCCGTTTTCTGACCCGATGGCGGATGGTCCGAC
MO00036-1        3041 ATGCACAGCCTGGTAGCTAACGGTGCTGATATTTTGGAACTGGGTGTGCCGTTTTCTGACCCGATGGCGGATGGTCCGAC
MO00036-2        3041 ATGCACAGCCTGGTAGCTAACGGTGCTGATATTTTGGAACTGGGTGTGCCGTTTTCTGACCCGATGGCGGATGGTCCGAC
MO00041-1        3041 ATGCACAGCCTGGTAGCTAACGGTGCTGATATTTTGGAACTGGGTGTGCCGTTTTCTGACCCGATGGCGGATGGTCCGAC
MO00049-1        3041 ATGCACAGCCTGGTGGCTAATGGTGCTGATATTTTGGAACTGGGTGTGCCGTTTTCTGACCCGATGGCGGATGGTCCGAC
MO0009-1         1098 ATGCATAGCCTGGTGGCTAATGGTGCTGATATTTTGGAACTGGGTGTGCCGTTTTCTGACCCGATGGCGGATGGTCCGAC
MO00045-1        1098 ATGCATAGCCTGGTGGCTAATGGTGCTGATATTTTGGAACTGGGTGTGCCGTTTTCTGACCCGATGGCGGATGGCCCGAC
2006-3950_Strep       --------------------------------------------------------------------------------
                                                      trpA

MO0003-2         3121 CATTCAGCGTGCGGCCGAAAGGGCGTTGGCAAATCATGTTTCTTTGCACGACGTATTGGAAACAGTACGCTTGTTCCGCC
MO0027-1         3121 CATTCAGCGTGCGGCCGAAAGGGCGTTGGCAAATCATGTTTCTTTGCACGACGTATTGGAAACAGTACGCTTGTTCCGCC
MO00027-2        3121 CATTCAGCGTGCGGCCGAAAGGGCGTTGGCAAATCATGTTTCTTTGCACGACGTATTGGAAACAGTACGCTTGTTCCGCC
MO0009-2         3121 CATTCAGCGTGCGGCCGAAAGGGCGTTGGCAAATCATGTTTCTTTGCACGACGTATTGGAAACAGTACGCTTGTTCCGCC
MO00029-1        3121 CATTCAGCGTGCGGCCGAAAGGGCGTTGGCAAATCATGTTTCTTTGCACGACGTATTGGAAACAGTACGCTTGTTCCGCC
MO00036-1        3121 CATTCAGCGTGCGGCCGAAAGGGCGTTGGCAAATCATGTTTCTTTGCACGACGTATTGGAAACAGTACGCTTGTTCCGCC
MO00036-2        3121 CATTCAGCGTGCGGCCGAAAGGGCGTTGGCAAATCATGTTTCTTTGCACGACGTATTGGAAACAGTACGCTTGTTCCGCC
MO00041-1        3121 CATTCAGCGTGCGGCCGAAAGGGCGTTGGCAAATCATGTTTCTTTGCACGACGTATTGGAAACAGTACGCTTGTTCCGCC
MO00049-1        3121 TATTCAACGTGCGGCTGAACGGGCGTTGACAAATCATGTTTCTTTGCACGACGTATTGGAAACAGTACGATTGTTCCGCC
MO0009-1         1178 CATTCAGCGTGCGGCCGAAAGGGCGTTGGCAAATCATGTTTCTTTGCACGACGTATTGGAAACAGTACGTTTGTTCCGCC
MO00045-1        1178 TATTCAACGTGCGGCTGAAAGGGCGTTGGCAAATCATGTTTCTTTGCGCGACGTATTGGAAACAGTACGCTTGTTCCGCC
2006-3950_Strep       --------------------------------------------------------------------------------
                                                      trpA

MO0003-2         3201 AAACCAATGATAAAACGCCGATTGTGTTAATGGGCTATTTGAACCCAGTACATAAAATGGGTTATAAAGCGTTTGCACAA
MO0027-1         3201 AAACCAATGATAAAACGCCGATTGTGTTAATGGGCTATTTGAACCCAGTACATAAAATGGGTTATAAAGCGTTTGCACAA
MO00027-2        3201 AAACCAATGATAAAACGCCGATTGTGTTAATGGGCTATTTGAACCCAGTACATAAAATGGGTTATAAAGCGTTTGCACAA
MO0009-2         3201 AAACCAATGATAAAACGCCGATTGTGTTAATGGGCTATTTGAACCCAGTACATAAAATGGGTTATAAAGCGTTTGCACAA
MO00029-1        3201 AAACCAATGATAAAACGCCGATTGTGTTAATGGGCTATTTGAACCCAGTACATAAAATGGGTTATAAAGCGTTTGCACAA
MO00036-1        3201 AAACCAATGATAAAACGCCGATTGTGTTAATGGGCTATTTGAACCCAGTACATAAAATGGGTTATAAAGCGTTTGCACAA
MO00036-2        3201 AAACCAATGATAAAACGCCGATTGTGTTAATGGGCTATTTGAACCCAGTACATAAAATGGGTTATAAAGCGTTTGCACAA
MO00041-1        3201 AAACCAATGATAAAACGCCGATTGTGTTAATGGGCTATTTGAACCCAGTACATAAAATGGGTTATAAAGCGTTTGCACAA
MO00049-1        3201 AAACTAATGATAAAACGCCGGTTGTGTTAATGGGCTATTTGAACCCCATACATAAAATGGGCTATCAGGCGTTTGCGCAA
MO0009-1         1258 AAACCAATGATAAAACGCCGATTGTGTTAATGGGCTATTTGAACCCAGTACATAAAATGGGTTATCAAGCGTTTGCACAA
MO00045-1        1258 AAACCAATGATAAAACGCCGGTTGTGTTAATGGGCTATTTGAACCCTGTACACAAAATGGGTTATCAGGCGTTTGCGCAA
2006-3950_Strep       --------------------------------------------------------------------------------
                                                      trpA

MO0003-2         3281 GCGGCTGCCGACGCGGGCGTAGATGGCGTGTTGACTGTGGATTCCCCGGTAGAAACCATCACCCCGCTGCATGACTCTTT
MO0027-1         3281 GCGGCTGCCGACGCGGGCGTAGATGGCGTGTTGACTGTGGATTCCCCGGTAGAAACCATCACCCCGCTGCATGACTCTTT
MO00027-2        3281 GCGGCTGCCGACGCGGGCGTAGATGGCGTGTTGACTGTGGATTCCCCGGTAGAAACCATCACCCCGCTGCATGACTCTTT
MO0009-2         3281 GCGGCTGCCGACGCGGGCGTAGATGGCGTGTTGACTGTGGATTCCCCGGTAGAAACCATCACCCCGCTGCATGACTCTTT
MO00029-1        3281 GCGGCTGCCGACGCGGGCGTAGATGGCGTGTTGACTGTGGATTCCCCGGTAGAAACCATCACCCCGCTGCATGACTCTTT
MO00036-1        3281 GCGGCTGCCGACGCGGGCGTAGATGGCGTGTTGACTGTGGATTCCCCGGTAGAAACCATCACCCCGCTGCATGACTCTTT
MO00036-2        3281 GCGGCTGCCGACGCGGGCGTAGATGGCGTGTTGACTGTGGATTCCCCGGTAGAAACCATCACCCCGCTGCATGACTCTTT
MO00041-1        3281 GCGGCTGCCGACGCGGGCGTAGATGGCGTGTTGACTGTGGATTCCCCGGTAGAAACCATCACCCCGCTGCATGACTCTTT
MO00049-1        3281 GCGGCTGCCGAGGCAGGCGTAGATGGCGTGTTGACCGTGGATTCTCCGGTAGAAACCATTGCGCCTTTGCATGAATCGTT
MO0009-1         1338 GCGGCTGCCGACGCGGGCGTAGATGGCGTGTTGACTGTGGATTCTCCGGTAGAAACCATTACCCCACTGCATGACTCGTT
MO00045-1        1338 GCGGCTGCCGAGGCAGGCGTTGATGGCGTGTTGACCGTAGATTCTCCGGTAGAAACCATTGCGCCTTTGCATGAGTCATT
2006-3950_Strep       --------------------------------------------------------------------------------
                                                      trpA
```

```
MO0003-2        3361 GAAAGCACAAGGTATTGATTGTATTTTCCTTATTGCGCCGACAACAACTGAAGAGCGTATTCAAACGATTGCCAAAGTAG
MO0027-1        3361 GAAAGCACAAGGTATTGATTGTATTTTCCTTATTGCGCCGACAACAACTGAAGAGCGTATTCAAACGATTGCCAAAGTAG
MO00027-2       3361 GAAAGCACAAGGTATTGATTGTATTTTCCTTATTGCGCCGACAACAACTGAAGAGCGTATTCAAACGATTGCCAAAGTAG
MO0009-2        3361 GAAAGCACAAGGTATTGATTGTATTTTCCTTATTGCGCCGACAACAACTGAAGAGCGTATTCAAACGATTGCCAAAGTAG
MO00029-1       3361 GAAAGCACAAGGTATTGATTGTATTTTCCTTATTGCGCCGACAACAACTGAAGAGCGTATTCAAACGATTGCCAAAGTAG
MO00036-1       3361 GAAAGCACAAGGTATTGATTGTATTTTCCTTATTGCGCCGACAACAACTGAAGAGCGTATTCAAACGATTGCCAAAGTAG
MO00036-2       3361 GAAAGCACAAGGTATTGATTGTATTTTCCTTATTGCGCCGACAACAACTGAAGAGCGTATTCAAACGATTGCCAAAGTAG
MO00041-1       3361 GAAAGCACAAGGTATTGATTGTATTTTCCTTATTGCGCCGACAACAACTGAAGAGCGTATTCAAACGATTGCCAAAGTAG
MO00049-1       3361 GAAAGCGCAAGGTATTGATTGTATTTTCCTTATTGCGCCAACGACAACTGAAGAGCGTATTCAAACGATTGCCAAAGTAA
MO0009-1        1418 GAAAGCGCAAGGTATTGATTGTATTTTCCTTATTGCGCCGACAACAACTGAAGAGCGTATTCAAACGATTGCCAAAGTAG
MO00045-1       1418 GAAAGCACAAGGTATTGATTGTATTTTCCTTATTGCACCAACGACAACTGAGGAGCGTATTCAAACGATTGCCAAAGTAG
2006-3950_Strep      --------------------------------------------------------------------------------
                                                        trpA

MO0003-2        3441 CCGGTGGTTTTGTGTATTATGTATCGCTCAAAGGTGTAACCGGTGCGGCAAGTTTGGATACTGAAGAAGTTTCGCGTAAA
MO0027-1        3441 CCGGTGGCTTTGTGTATTATGTATCGCTCAAAGGTGTAACCGGTGCGGCAAGTTTGGATACTGAAGAAGTTTCGCGTAAA
MO00027-2       3441 CCGGTGGTTTTGTGTATTATGTATCGCTCAAAGGTGTAACCGGTGCGGCAAGTTTGGATACTGAAGAAGTTTCGCGTAAA
MO0009-2        3441 CCGGTGGTTTTGTGTATTATGTATCGCTCAAAGGTGTAACCGGTGCGGCAAGTTTGGATACTGAAGAAGTTTCGCGTAAA
MO00029-1       3441 CCGGTGGTTTTGTGTATTATGTATCGCTCAAAGGTGTAACCGGTGCGGCAAGTTTGGATACTGAAGAAGTTTCGCGTAAA
MO00036-1       3441 CCGGTGGTTTTGTGTATTATGTATCGCTCAAAGGTGTAACCGGTGCGGCAAGTTTGGATACTGAAGAAGTTTCGCGTAAA
MO00036-2       3441 CCGGTGGTTTTGTGTATTATGTATCGCTCAAAGGTGTAACCGGTGCGGCAAGTTTGGATACTGAAGAAGTTTCGCGTAAA
MO00041-1       3441 CCGGTGGTTTTGTGTATTATGTATCGCTCAAAGGTGTAACCGGTGCGGCAAGTTTGGATACTGAAGAAGTTTCGCGTAAA
MO00049-1       3441 CCGGCGGTTTTGTGTATTATGTATCGCTCAAAGGCGTGACCGGTGCAGCAAGTTTGGATACTGAAGAAGTTTCGCGTAAA
MO0009-1        1498 CCGGTGGCTTTGTGTATTATGTATCGCTCAAAGGTGTAACCGGTGCGGCAAATTTGGATACTGAAGAAGTTTCGCGTAAA
MO00045-1       1498 CCGGCGGTTTTGTGTATTATGTATCGCTCAAAGGTGTAACCGGTGCGGCAAGTTTGGACACTGAAGAAGTTTCGCGTAAA
2006-3950_Strep      --------------------------------------------------------------------------------
                                                        trpA

MO0003-2        3521 ATAGAGCTTTTGCGCAAATACATCGATATTCCGATTGGTGTCGGCTTCGGCATCAATAATGCGGAGAGTGCGCGTAAGAT
MO0027-1        3521 ATAGAGCTTTTGCGCAAATATATTGATATTCCGATCGGTGTCGGCTTCGGCATTAATAATGCGGAGAGTGCGCGTAAGAT
MO00027-2       3521 ATAGAGCTTTTGCGCAAATACATCGATATTCCGATTGGTGTCGGCTTCGGCATCAATAATGCGGAGAGTGCGCGTAAGAT
MO0009-2        3521 ATAGAGCTTTTGCGCAAATACATCGATATTCCGATTGGTGTCGGCTTCGGCATCAATAATGCGGAGAGTGCGCGTAAGAT
MO00029-1       3521 ATAGAGCTTTTGCGCAAATACATCGATATTCCGATTGGTGTCGGCTTCGGCATCAATAATGCGGAGAGTGCGCGTAAGAT
MO00036-1       3521 ATAGAGCTTTTGCGCAAATACATCGATATTCCGATTGGTGTCGGCTTCGGCATCAATAATGCGGAGAATGCGCGTAAAAT
MO00036-2       3521 ATAGAGCTTTTGCGCAAATACATCGATATTCCGATTGGTGTCGGCTTCGGCATCAATAATGCGGAGAATGCGCGTAAAAT
MO00041-1       3521 ATAGAGCTTTTGCGCAAATACATCGATATTCCGATTGGTGTCGGCTTCGGCATCAATAATGCGGAGAGTGCGCGTAAGAT
MO00049-1       3521 ATAGAGCTTTTGCGCAAATACATTGATATTCCGATTGGTGTCGGCTTCGGCATCAATAATGCGGAGAGCGCGCGTAAGAT
MO0009-1        1578 ATAGAACTTTTGCGCAAATATATTGATATTCCGATCGGTGTCGGCTTCGGCATTAATAATGCGGAGAGTGCGCGTAAGAT
MO00045-1       1578 ATAGAGCTTTTGCGCAAATACATTGATATTCCGATTGGTGTCGGCTTCGGCATTAATAATGCGGAGAGTGCGCGTAAGAT
2006-3950_Strep      --------------------------------------------------------------------------------
                                                        trpA

MO0003-2        3601 TGGTGCGGTGGCGGATGCCGTTATTGTCGGCAGCCGTATCGTCAAAGAGATTGAAAGCCATGCGGGTAGCGAGGCTGAAG
MO0027-1        3601 TGGCGCGGTAGCGGATGCCGTTATTGTCGGCAGCCGTATCGTCAAAGAGATTGAAAGCCACGCAGGTAGCGAGGCTGAGG
MO00027-2       3601 TGGTGCGGTGGCGGATGCCGTTATTGTCGGCAGCCGTATCGTCAAAGAGATTGAAAGCCATGCGGGTAGCGAGGCTGAAG
MO0009-2        3601 TGGTGCGGTGGCGGATGCCGTTATTGTCGGCAGCCGTATCGTCAAAGAGATTGAAAGCCATGCGGGTAGCGAGGCTGAAG
MO00029-1       3601 TGGTGCGGTGGCGGATGCCGTTATTGTCGGCAGCCGTATCGTCAAAGAGATTGAAAGCCATGCGGGTAGCGAGGCTGAAG
MO00036-1       3601 TGGTGCGGTAGCGGATGCCGTTATTGTCGGCAGCCGTATCGTCAAAGAGATTGAAAGCCACGCAGGTAGCGAGGCCGAAG
MO00036-2       3601 TGGTGCGGTAGCGGATGCCGTTATTGTCGGCAGCCGTATCGTCAAAGAGATTGAAAGCCACGCAGGTAGCGAGGCCGAAG
MO00041-1       3601 TGGTGCGGTGGCGGATGCCGTTATTGTCGGCAGCCGTATCGTCAAAGAGATTGAAAGCCATGCGGGTAGCGAGGCTGAAG
MO00049-1       3601 TGGAGCGGTGGCGGATGCAGTTATTGTCGGCAGCCGTATCGTCAAAGAGATTGAAAGTCACGCGGGTAGCGAGGCTGAGG
MO0009-1        1658 TGGCGCGGTGTCGGATGCTGTTATTGTCGGCAGCCGTATCGTCAAAGAGATTGAAAGCCACGCAGGTAGCGAGGCCGAAG
MO00045-1       1658 TGGTGCGGTGGCGGATGCCGTTATTGTCGGCAGTCGTATCGTCAAAGAGATTGAAAGCCACGCGGGTAGCGAGGCTGAAG
2006-3950_Strep      --------------------------------------------------------------------------------
                                                        trpA

MO0003-2        3681 CAGTAGGCGCGTTGGTTAAAGAGTTGAAAGACGCGATTCGCTAAATTTTTTCATTCCATTGTTTC
MO0027-1        3681 CGGTAGGCGTGTTGGTTAAAGAGTTGAAAGACGCGATTCGCTAAATTTTTT-ATTCCATTATTTC
MO00027-2       3681 CAGTAGGCGCGTTGGTTAAAGAGTTGAAAGACGCGATTCGCTAAATTTTTTCATTCCATTGTTTC
MO0009-2        3681 CAGTAGGCGCGTTGGTTAAAGAGTTGAAAGACGCGATTCGCTAAATTTTTTCATTCCATTGTTTC
MO00029-1       3681 CAGTAGGCGCGTTGGTTAAAGAGTTGAAAGACGCGATTCGCTAAATTTTTTCATTCCATTGTTTC
MO00036-1       3681 CGGTAGGCGTGTTGGTTAAAGAGTTGAAAGACGCGATTCGCTAAATTTTTT-ATTCCATTATTTC
MO00036-2       3681 CGGTAGGCGTGTTGGTTAAAGAGTTGAAAGACGCGATTCGCTAAATTTTTT-ATTCCATTATTTC
MO00041-1       3681 CAGTAGGCGCGTTGGTTAAAGAGTTGAAAGACGCGATTCGCTAAATTTTTTCATTCCATTGTTTC
MO00049-1       3681 CAGTAGGCGCGTTAGTTAAAGAGTTGAAAGACGCAATTTGCTAAATTTTTCCATTCCATTGTTTC
MO0009-1        1738 CGGTAGGCGTGTTGGTTAAAGAGTTGAAAGACGCGATTCGCTAATATTTTTTATTCCATTATTTC
MO00045-1       1738 CAGTAGGCGTGTTGGTTAAAGAGTTGAAAGACGCGATTCGCTAAATTTTTCCATTCCATTGTTTC
2006-3950_Strep      -----------------------------------------------------------------
                                        trpA
```

Supplemental figure 3. Alignment of all *N. subflava* at the integration site of *msr(D)* with up and downstream regions and integrated fragment of the MEGA element (region 2006-3950). Indicated are: *ihfA*, *msr(D)* and *trpA* in grey bar with start and top codon indicated in cyan background, *mefA* (fragment 880-1218) grey striped bar, regulatory sequence factor MYLIMF and DUS factor in yellow box, integration site with green horizontal line.
