# Supplementary material for: WGS of Commensal Neisseria Reveals Acquisition of a New Ribosomal Protection Protein (MsrD) as a Possible Explanation for High Level Azithromycin Resistance in Belgium
Source: Pathogens. 2021 Mar 23;10(3):384. doi: 10.3390/pathogens10030384 (PMC8005064; doi:10.3390/pathogens10030384)
Supplement: Supplementary file 1 [file pathogens-10-00384-s001.zip › Supplemental_Figure_4.pdf]

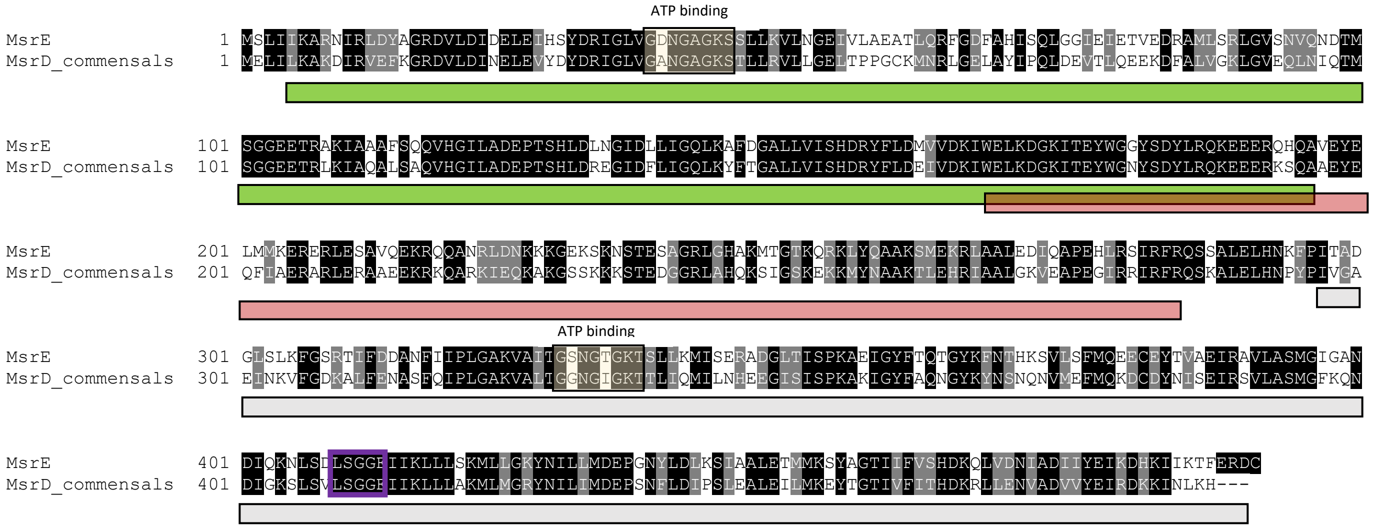

Supplemental figure 4. Alignment of msrE and msrD, with ATP binding domains, ABC1 domain (green bar), ABC2 domain (grey bar), P site tRNA interaction motif (PtIM, formerly called ABC_tran_Xtn, red bar) and signature sequence (purple box).
