# Supplementary material for: WGS of Commensal Neisseria Reveals Acquisition of a New Ribosomal Protection Protein (MsrD) as a Possible Explanation for High Level Azithromycin Resistance in Belgium
Source: Pathogens. 2021 Mar 23;10(3):384. doi: 10.3390/pathogens10030384 (PMC8005064; doi:10.3390/pathogens10030384)

Supplemental figure 5. Acquisition of TetM (blue) in *N. gonorrhoeae* (MO0023/1) (A) and *N. subflava* (MO0009/1) (B). Genome assembly based on illumina only reads (A) and hybrid illumina – MinION reads (B). This clearly shows that *tetM* is plasmid-mediated in *N. gonorrhoeae*, but in *N. sublfava* this gene is incorporated into the chromosome.

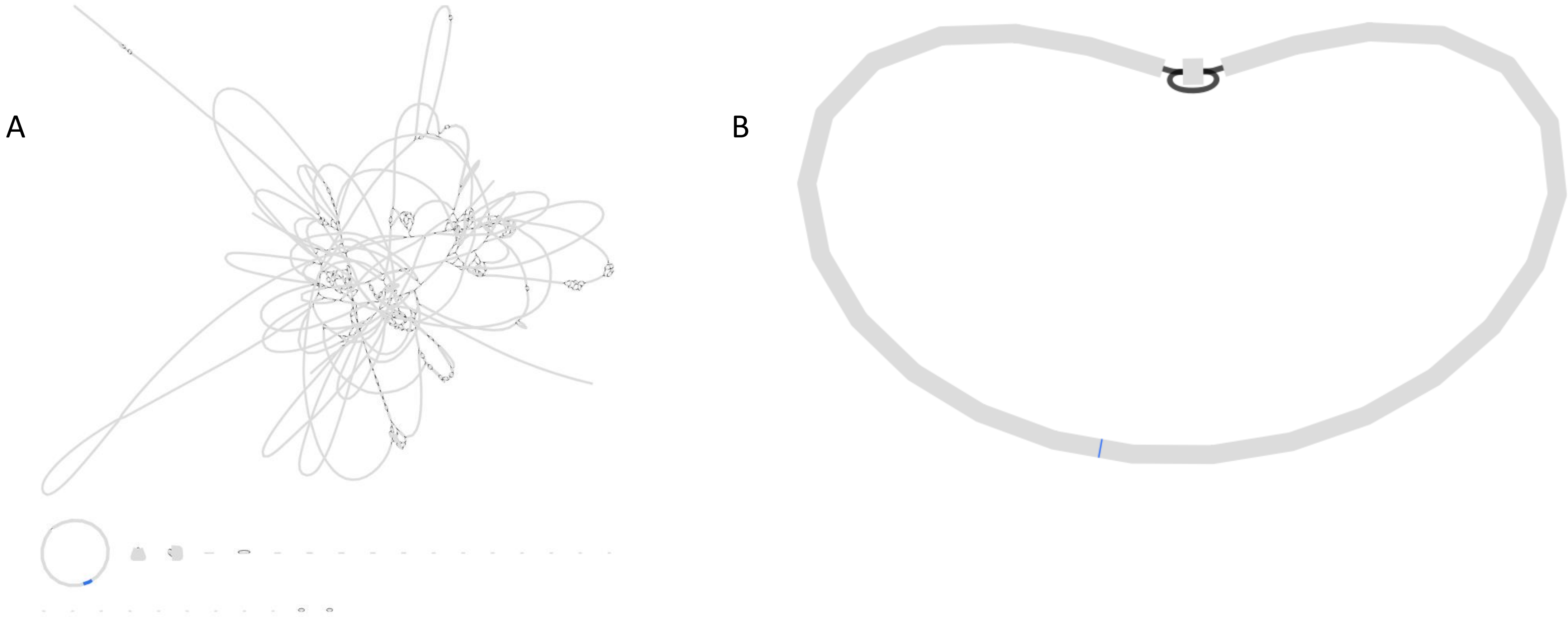

Supplement: Supplementary file 1 [file pathogens-10-00384-s001.zip › Supplemental_Figure_5_II.pdf]
