# Supplementary material for: WGS of Commensal Neisseria Reveals Acquisition of a New Ribosomal Protection Protein (MsrD) as a Possible Explanation for High Level Azithromycin Resistance in Belgium
Source: Pathogens. 2021 Mar 23;10(3):384. doi: 10.3390/pathogens10030384 (PMC8005064; doi:10.3390/pathogens10030384)
Supplement: Supplementary file 1 [file pathogens-10-00384-s001.zip › Supplemental_Table_1.pdf]

Sample	Genome Size (bp)	No contigs	N 50	No CDS	GC (%)	biosample accession
MO00014/1	2093282	76	71108	2045	45.83	SAMN18012326
MO00020/1	2154157	90	78434	2096	50.66	SAMN18012327
MO00023/1	2148913	84	57100	2114	49.12	SAMN18012328
MO00038/1	2155886	76	67433	2113	52.46	SAMN18012329
MO00044/1.3	2123854	150	27442	2055	52.53	SAMN18012330
MO0003/1	2116072	58	79361	1986	49.55	SAMN18012331
MO00012/1	2325937	91	92210	2181	49.14	SAMN18012332
MO00015/1	2248695	74	82127	2127	48.92	SAMN18012333
MO00015/2	2278599	90	82313	2161	49.02	SAMN18012334
MO00021/1	2263795	93	66342	2125	49.17	SAMN18012335
MO00018/1	2475065	20	529403	2268	50.56	SAMN18012336
MO00032/1	2475385	20	529474	2273	52.77	SAMN18012337
MO00012/2	2767239	89	64117	2516	50.58	SAMN18012338
MO00029/2	2819881	48	168641	2528	48.74	SAMN18012339
MO00018/2	2762606	51	212926	2535	51.30	SAMN18012340
MO0003/2	2222383	11	496641	2102	50.92	SAMN18012341
MO0009/1	2332010	45	128194	2206	52.61	SAMN18012342
MO0009/2	2261404	17	422373	2137	49.22	SAMN18012343
MO00027/1	2247288	43	84192	2154	52.64	SAMN18012344
MO00027/2	2374291	43	194400	2277	51.54	SAMN18012345
MO00029/1	2194345	10	1186504	2018	51.60	SAMN18012346
MO00036/1	2291869	23	408621	2187	50.99	SAMN18012347
MO00036/2	2291659	21	408984	2187	52.76	SAMN18012348
MO00041/1	2221987	20	460972	2102	51.54	SAMN18012349
MO00045/1	2200796	23	276433	2061	49.03	SAMN18012350
MO00049/1	2424202	31	185666	2326	52.42	SAMN18012351
